# Selecting a preculture strategy for improving biomass and astaxanthin productivity of *Chromochloris zofingiensis*

**DOI:** 10.1007/s00253-023-12873-x

**Published:** 2024-01-10

**Authors:** Yuxin Wang, Jia Wang, Shufang Yang, Qingping Liang, Ziqiang Gu, Ying Wang, Haijin Mou, Han Sun

**Affiliations:** 1 College of Food Science and Engineering, Ocean University of China, Qingdao, 266003 China; 2 Institute for Advanced Study, Shenzhen University, Shenzhen, 518060 China; 3 Marine Science research Institute of Shandong Province, Qingdao, 266003 China

**Keywords:** Preculture, *Chromochloris zofingiensis*, Astaxanthin, Carbon metabolism, High-density production

## Abstract

**Abstract:**

*Chromochloris zofingiensis* is a potential source of natural astaxanthin; however, its rapid growth and astaxanthin enrichment cannot be achieved simultaneously. This study established autotrophic, mixotrophic, and heterotrophic preculture patterns to assess their ameliorative effect on the *C. zofingiensis* heterotrophic growth state. In comparison, mixotrophic preculture (MP) exhibited the best improving effect on heterotrophic biomass concentration of *C. zofingiensis* (up to 121.5 g L^−1^) in a 20 L fermenter, reaching the global leading level. The astaxanthin productivity achieved 111 mg L^−1^ day^−1^, 7.4-fold higher than the best record. The transcriptome and ^13^C tracer-based metabolic flux analysis were used for mechanism inquiry. The results revealed that MP promoted carotenoid and lipid synthesis, and supported synthesis preference of low unsaturated fatty acids represented by C18:1 and C16:0. The MP group maintained the best astaxanthin productivity via mastering the balance between increasing glucose metabolism and inhibition of carotenoid synthesis. The MP strategy optimized the physiological state of *C. zofingiensis* and realized its heterotrophic high-density growth for an excellent astaxanthin yield on a pilot scale. This strategy exhibits great application potential in the microalgae-related industry.

**Key points:**

• *Preculture strategies changed carbon flux and gene expression in C. zofingiensis*

•* C. zofingiensis realized a high-density culture with MP and fed-batch culture (FBC)*

• *Astaxanthin productivity achieved 0.111 g L*^−1^ *day*^−1^ *with MP and FBC*

**Graphical Abstract:**

**Figure:**
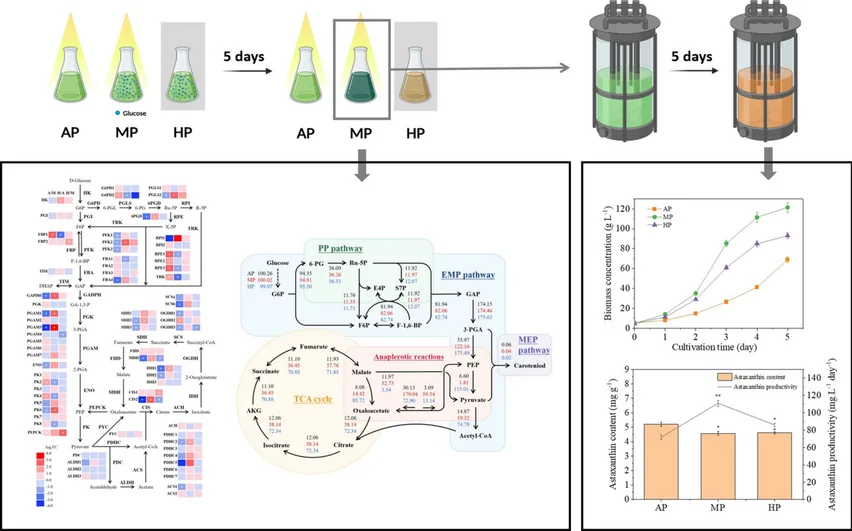

**Supplementary Information:**

The online version contains supplementary material available at 10.1007/s00253-023-12873-x.

## Introduction

Microalgae are known as promising cell factories for synthesizing multiple value-added metabolites, including storage lipids and astaxanthin. Astaxanthin exhibits a broad market prospect in aquaculture, nutraceuticals, and foods, and has been emerging into the limelight with exceptional antioxidant activity and other physiological effects (Sun et al. 2023). Microalgal astaxanthin, consistent with the structure of astaxanthin in aquatic animals, is eminently absorbable and available for the human body (Mussagy et al. 2019). Therefore, cultivating microalgal organisms now becomes a feasible approach for sustainable astaxanthin provision. *Haematococcus pluvialis* is the richest microalgal source and the major commercial producer of natural astaxanthin (Yang et al. 2023). However, the highest reported productivity of astaxanthin was only 18.1 mg L^−1^ day^−1^ limited by its autotrophic property (Mota et al. 2022). *Chromochloris zofingiensis*, another species of green algae with the capacity for astaxanthin synthesis, can grow fast autotrophically, mixotrophically, and even heterotrophically (Liu et al. 2013; Zhao et al. 2018). *C. zofingiensis*-derived astaxanthin accounts for 70% of total secondary carotenoids. It exists in 3S, 3’S, and almost all-*trans* form and is esterified on its one or both hydroxyl groups with various fatty acids; these characteristics endows it with excellent antioxidant bioactivity and stability (Liu et al. 2014). Therefore, this species has been reckoned to be the potential alternative of *H. pluvialis* as a producer of natural astaxanthin. Its heterotrophic growth can achieve faster growth speed, higher cell density, and shorter fermentation period through avoiding the energy deficiency caused by insufficient illumination, and can undoubtably serve as an alternative producing method of natural astaxanthin (Fang et al. 2019).

The efficient production of astaxanthin hinges on its intracellular accumulation and high cell-density growth. Astaxanthin synthesis was identified as a protective response to photo-oxidative stress stimulated by the over-reduction in the photosynthetic electron transport chain (Chen et al. 2022). Consequently, the astaxanthin content of heterotrophically cultured *C. zofingiensis* is inevitably inferior to that of the autotrophic mode. To compensate for the productivity reduction, the current study of *C. zofingiensis* emphasizes reaching the maximal biomass yield via a high-density cultivation technology, which is the primary purpose of most heterotrophic fermentation techniques. Unfortunately, for wild *C. zofingiensis*, or many other autotrophic microalgae, a sudden condition change may weaken the cell viability, and the biomass in early generations after inoculation into the heterotrophic condition is not satisfactory, which is known as cause of the lag phase (Kim et al. 2021). To eliminate this bottleneck, numerous culture strategies aiming at optimizing heterotrophic medium composition have been developed (Ip and Chen 2005; Jiang et al. 2015; Wang et al. 2023b). Chen et al. (2022) designed a two-step strategy to realize the record-setting biomass and astaxanthin yields (235.4 g L^−1^ and 0.318 g L^−1^, respectively). However, this complicated process requires multistep operations and a cultivation period as long as 21 days.

Preculture is an efficient strategy proposed to help cells acclimate to a drastic condition transformation. A preculture condition is usually provided in a transitional state between the original and target conditions; the usage of this intermediate step can improve the physiological state of cells that were first inoculated into the target condition, thus accelerating cell growth and shortening the lag phase (Kim et al. 2021; Sun et al. 2021b). Establishing a simple and feasible preculture strategy is the standard approach to promote the yield and product quality in the microbial-based industry, and there is no exception that it also has potential improvement effects on the astaxanthin production of *C. zofingiensis.* This study explored the impacts of different preculture strategies on improving the heterotrophic high-density growth of *C. zofingiensis*, and investigated the further application possibility of them in industrial astaxanthin production.

*C. zofingiensis* cells were precultured under autotrophic, mixotrophic, and heterotrophic modes and then transferred to heterotrophic culture. After each strategy, the cells’ growth characteristics, composition, and astaxanthin productivity were analyzed. Since autotrophic preculture is the simulation of wild photoautotrophic strain, this group can also be reckoned as the control group to assess the impact of mixotrophic and heterotrophic preculture strategies. Lipid carbon flux in cells was explored by establishing a carbon distribution model. The transcriptome was analyzed to investigate the effect of different strategies on astaxanthin synthesis. ^13^C tracer-based metabolic flux analysis (^13^C-MFA) explored global relationships between carotenoid biosynthesis and central carbon metabolism. Finally, a fed-batch culture (FBC) mode was established to verify the effect of preculture strategies on *C. zofingiensis* astaxanthin productivity on an amplified scale*.*

## Material and methods

### Batch culture of *C. zofingiensis*

*C. zofingiensis* (strain ATCC 30412, from the American Type Culture Collection) was cultivated in 250-mL Erlenmeyer flasks containing autoclaved BG-11 medium (NaNO_3_ 1.5 g L^−1^, K_2_HPO_4_ 40 mg L^−1^, Na_2_CO_3_ 20 mg L^−1^, H_3_BO_3_ 2.86 mg L^−1^, MgSO_4_·7H_2_O 75 mg L^−1^, CaCl_2_·2H_2_O 36 mg L^−1^, EDTANa_2_ 1 mg L^−1^, MnCl_2_·4H_2_O 1.81 mg L^−1^, citric acid 6 mg L^−1^, ammonium ferric citrate 6 mg L^−1^, ZnSO_4_·7H_2_O 0.22 mg L^−1^, Na_2_MoO_4_·2H_2_O 0.39 mg L^−1^, CuSO_4_·5H_2_O 0.08 mg L^−1^, pH 7.2). The Erlenmeyer flasks were put in a 22 °C constant temperature shaker with a speed of 140 rpm and illuminated by constant white light of 50 μE m^−2^ s^−1^. This condition was also adopted to obtain cells with autotrophic preculture (AP), collectively called the AP group hereinafter. The mixotrophic preculture (MP) group used a medium with 5 g L^−1^ glucose as the extra carbon source (Sun et al. 2021a), the same nutritional condition without light was set in the heterotrophic preculture (HP) group. During each generation, *C. zofingiensis* was cultivated for 5 days and then inoculated into new media at 10% (v/v). After three generations of preculture, the cells were inoculated into the same heterotrophic condition.

### Exponential fed-batch culture of *C. zofingiensis*

Exponential fed-batch culture was established in a 20 L fermenter; the temperature was set at 22 °C, the pressure was set at 0.05 MPa, the rotation speed was initially set at 100 rpm and gradually increased to 600 rpm, and the dissolved oxygen was set at 40% and gradually decreased to 0. Based on previous research (Sun et al. 2021a), the mass ratio of glucose, nitrate, and phosphate in the designed medium was set as 23:10:1 to maximize biomass concentration; and other conditions were the same as those in batch heterotrophic culture. The substrate and energy consumption of *C. zofingiensis* for cell maintenance was assumed to be low in the exponential growth phase, and extracellular products were rare. The FBC model was established as (Sun et al. 2020a)

1 $${V}_{FS}=\mu {X}_0\exp \left(\mu t\right){V}_0/{Y}_{XS}{C}_{FS}$$

where *Y*_*XS*_ represented the biomass yield on the substrate, *V*_*FS*_ was the feeding rate (mL d^−1^), and *V*_0_ was the initial volume (mL). *C*_*FS*_ represented the nutrient concentration (g L^−1^); for example, *C*_*FG*_ was the glucose concentration in the feeding medium, and *C*_*FN*_ and *C*_*FP*_ were that of nitrate and phosphate.

### Model development of carbon partitioning in *C. zofingiensis*

A carbon-partitioning model was contrived based on an existing study (Ryu et al. 2018). Nutrients in the medium are assimilated into proteins and carbohydrates after absorption. Proteins are partially converted to carbohydrates, lipids, and other complex biomolecules. Carbohydrates were partially reallocated to lipids. Therefore, carbon flux can be streamlined into two specific fluxes, separately leading to lipid synthesis. Based on these assumptions, the model can be established as follows:

2 $$d{Q}_P/ dt={Y}_{PX}\mu {X}_0\exp \left(\mu t\right)-{D}_P{Q}_{P0}\exp \left({\mu}_Pt\right)$$

3 $$d{Q}_C/ dt={Y}_{CX}\mu {X}_0\exp \left(\mu t\right)-{\alpha}_C{Q}_{C0}\exp \left({\mu}_Ct\right)$$

4 $$d{Q}_L/ dt=\left({\mu}_C/{\mu}_L\right){\alpha}_C{Q}_{C0}\exp \left({\mu}_Ct\right)+\left({\mu}_P/{\mu}_L\right){D}_P{Q}_{P0}\exp \left({\mu}_Pt\right)$$

where *Q*_*P*_, *Q*_*C*_, and *Q*_*L*_ were the concentrations of protein, carbohydrate, and lipid (g L^−1^); *t* was the cultivation time (h); *μ*, *μ*_*P*_, *μ*_*C*_, and *μ*_*L*_, respectively, represented specific rates of cell growth, protein, carbohydrate, and lipid (h^−1^). *Y*_*PX*_ and *Y*_*CX*_ were yields of protein and carbohydrate on biomass concentration (g g^−1^), *X*_0_ was the initial biomass concentration (g L^−1^), and *D*_*P*_ and *α*_*C*_ were defined as the conversion rates of protein and carbohydrate (h^−1^). Since the specific rate was influenced by protein content, it could be described as follows:

5 $$\mu ={\mu}_{max}\left(1-{C}_P/{C}_{P,\mathit{\max}}\right)$$

6 dX/dt=μX

where *C*_*P*_ was the protein concentration (g L^−1^) and *C*_*P, max*_ represented the maximum of it (g L ^−1^). *μ*_*P*_, *μ*_*C*_, and *μ*_*L*_ can also be described by Eq. (5).

### Differential gene expression analysis

Total RNA was extracted using the plant RNA extraction kit (TaKaRa, Tokyo, Japan), and contaminating DNA was removed with RNase-free DNase I (TaKaRa, Tokyo, Japan). RNA concentration and quality were analyzed by Agilent 2100 Bioanalyzer (Agilent Technologies, Santa Clara, CA, USA). After poly-A containing mRNA molecules were purified using poly-T oligo-attached magnetic beads, the mRNA was fragmented into small pieces and copied into first-strand cDNA, followed by second-strand cDNA synthesis using DNA polymerase I and RNase H. These cDNA fragments then had the addition of a single “A” base and subsequent ligation of the adapter. The products were purified and enriched with PCR amplification. The PCR yield was quantified by Invitrogen Qubit assay (Thermo Fisher, Waltham, MA, USA), and samples were pooled together to make a single-strand DNA circle (ssDNA circle) to form the final library. DNA nanoballs (DNBs) were generated with the ssDNA circle and single-end read of 50 bp through on the BGISEQ-500 platform (BGI, Guangdong, China) for data analysis.

Differential gene expression was analyzed using a previous study (Mao et al. 2020). RSEM software (Li and Dewey 2011) was used to calculate gene expression levels and R software (R Core Team [2020]; R: A language and environment for statistical computing, R Foundation for Statistical Computing, Vienna, Austria. URL https://www.R-project.org/.) was used to perform statistics (Roth et al. 2017). Pearson correlation between all samples was calculated using cor; and PCA analysis was performed with all samples using princomp. Identified DEGs (differentially expressed genes) between samples were clustered with Deseq2 software (http://biocounductor.org/packages/release/bioc/html/DESg2.html) (Love et al. 2014) using the parameters as fold change ≥ 2.00 and adjusted *p* value (*Padj*) ≤ 0.05.

### ^13^C tracer-based metabolic flux analysis

^13^C-MFA of MP cells was computed by designing biomass reaction with the biomass components as reference. The content of each nucleotide was quoted from published literature (Roth et al. 2017). Samples were derivatized with *N*-*tert*-butyldimethylsilyl-*N*-methyltrifluoroacetamide and analyzed by GC–MS (7890B-5977B, Agilent, Santa Clara, CA, USA). CO_2_ statistical weight was analyzed by 100% U-^13^C glucose and removed to modify the labeled amino acid abundance. The metabolic flux was set regarding a previous study (Sun et al. 2020a). Rates of reaction paths were calculated by Metran coding software (Zhao et al. 2015).

### Determination of biomass and constituent contents

Gravimetry was used to monitor biomass growth, which was expressed as the ratio of the dry weight of cells to the volume of culture fluid (g L^−1^). One milliliter of culture fluid was centrifuged at 5000 *g*. The sediment pellets were filtered on pre-weighed filter paper and thoroughly dried in the 60 °C oven. After that, the samples were cooled and weighed.

Lipid content was determined by sulfo-phospho-vanillin assays (Zhao et al. 2018). The culture fluid was heated to 100 °C with concentrated sulfuric acid. Vanillin phosphate was added into the mixture and shaken at 37 °C for 15 min. Absorbance was measured at 530 nm and converted into total lipid content by comparison with a standard curve made of commercially available edible oil as standard.

Protein content was determined using a BCA kit (#5000002; Bio-Rad Laboratories Inc., Hercules, CA, USA). One milliliter of culture fluid was centrifuged at 12,000 *g*, and the cell pellets were hydrolyzed with 2 M sodium hydroxide in 80 °C incubation. The mixture was centrifuged with 0.9 mL of water twice to collect all supernatants.

Carbohydrate content was determined by the phenol sulfuric acid method (Ma et al. 2016). The culture fluid was centrifuged at 5000 *g*. The sediment was washed twice and then incubated with glacial acetic acid. Then the mixture was centrifuged with acetone at 3500 *g*. The residue was dissolved in 4 M trifluoroacetic acid and boiled in a water bath. After centrifugation at 10,000 *g*, the supernatant was heated with phenol sulfate for 20 min. The absorbance at 490 nm was measured and converted into content by comparison with a glucose standard curve.

To determinate pigment composition, lyophilized cell powder was pulverized in liquid nitrogen, extracted with 99.9% methanol, and then incubated at 45 °C in darkness. The mixtures were centrifuged at 12,000 *g*, then pigment contents in the supernatant were determined by photometry as absorbance at 470, 652.4, and 665.2 nm, respectively. The pigment concentrations were calculated based on a previous study (Chokshi et al. 2017).

### Determination of astaxanthin content in C. zofingiensis

The ground powder was mixed into anhydrous ethanol and centrifugated at 3000 *g* to collect the supernatant; repeat adding anhydrous ethanol to the sediment and centrifuge until it turned white. The supernatants were collected together and centrifuged at 12,000 *g*. Then the supernatant was dried by nitrogen blowing. One milliliter of of anhydrous ethanol was added to redissolve the pigment. The whole process was carried out in dark and low-temperature condition.

Astaxanthin content in cells was analyzed by high-performance liquid chromatography (HPLC) (Waters 2695, Milford, MA, USA) equipped with a CAPCELL PAK C18 5 μm column (4.6 × 250 mm) (Shiseido, Tokyo, Japan) and a photodiode array detector. The operational parameters were based on a pervious study (Sun et al. 2020a).

### Determination of fatty acid composition in C. zofingiensis

Biomass was extracted using chloroform and methanol. Sodium chloride (0.75%) was added to the mixture and centrifuged at 5000 g for 5 min. The chloroform layer was esterified to fatty acid methyl esters (FAMEs) by incubation with 1% sulfuric acid in methanol. FAMEs were analyzed using a gas chromatography–mass spectrometer (GC-MS) (Shimadzu, Kyoto, Japan) equipped with an Rtx-2330 capillary column (Restek, Guangzhou, China). Helium was used as the carrier gas. The operational parameters were set according to a previous study (Liu et al. 2016).

### Determination of amino acids in C. zofingiensis

Cell pellets were washed twice and pelleted by centrifugation at 5000 *g* for 5 min. The pellets were hydrolyzed with 6 M hydrochloric acid at 110 °C for 12 h. The amino acid contents of hydrolysates were analyzed using an LC20AD-API TRAP 3200MD HPLC-MS/MS system (Shimadzu Corp., Kyoto, Japan) (Wang et al. 2023b).

### Statistical analysis

All experiments were conducted in at least three biological replicates to ensure reproducibility, and data were recorded as means value ± SD. Data were statistically analyzed with SPSS software (IBM Corp. Released 2019, IBM SPSS Statistics for Windows, Version 26.0. Armonk, NY: IBM Corp) and a *p*-value < 0.05 was recognized to be statistically significant. One-way analysis of variance (ANOVA) with post hoc multiple-comparison least significant difference (LSD) tests was used to detect significant differences.

## Results

### Effect of preculture on *C. zofingiensis* growth and carbon partitioning

**Fig. 1:**
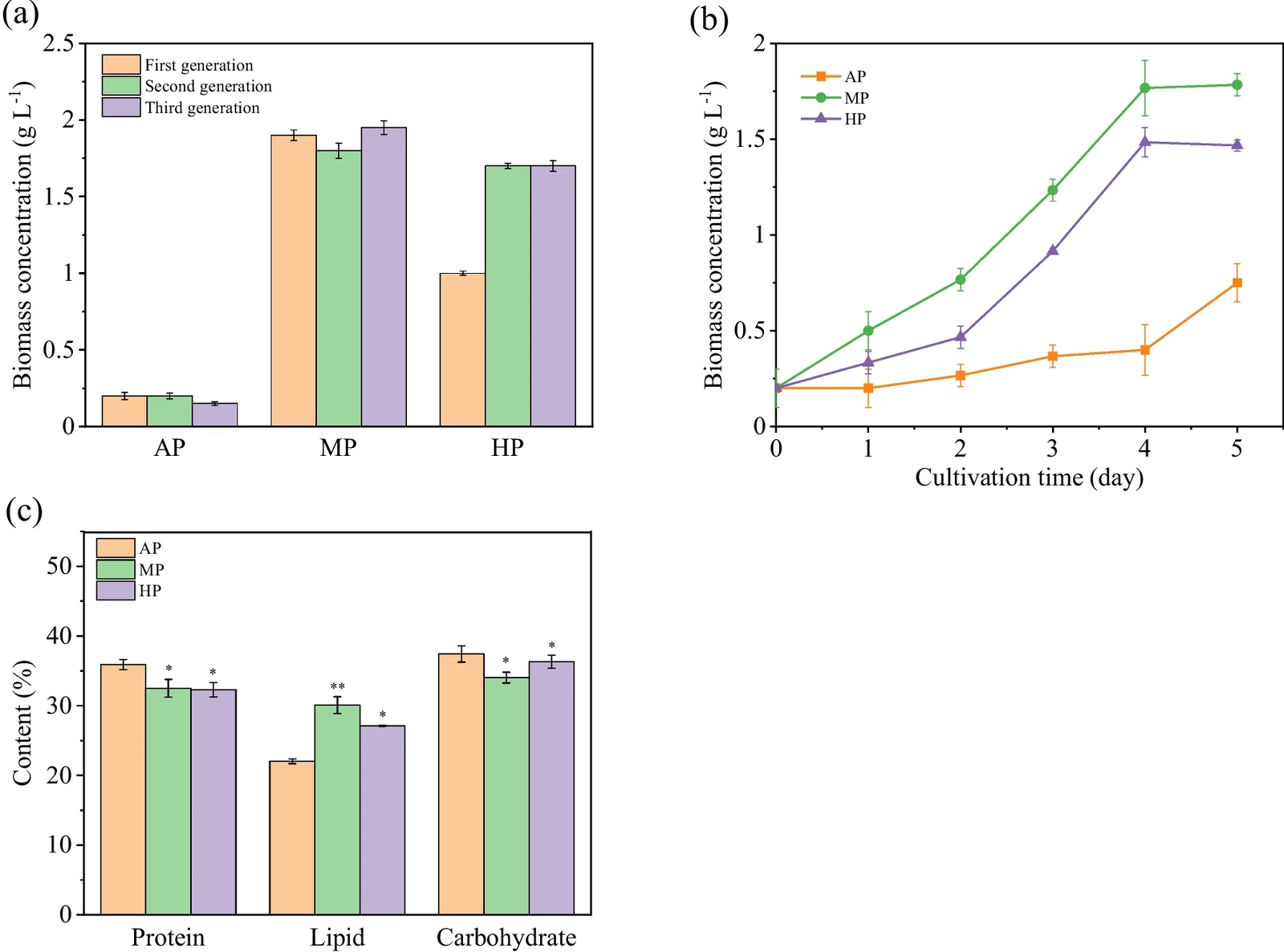
Biomass concentration after preculture (**a**); growth profile in heterotrophic batch culture (**b**); and protein, carbohydrate, and lipid contents (**c**) of *C. zofingiensis* in three preculture groups. Note: the value bar with ns means the difference between the corresponding group and the AP group is not significant, and asterisk denotes statistically significant differences * *p* < 0.05; ** *p* < 0.01

At first, *C. zofingiensis* was precultured under different strategies, among which MP and HP both demonstrated the capacity to promote biomass accumulation from the first preculture generation (Fig. 1a). When inoculated into heterotrophic condition, the dynamic change of cell biomass in the exponential growth phase was monitored. Figure 1b shows that cells after different preculture strategies bred similar grow profile trends but with apparent disparities in the final biomass and growth rate. *C. zofingiensis* maintained the highest growth rate after MP, with the final biomass of 1.78 g L^−1^, doubling that after AP (0.75 g L^−1^). The biomass of the HP group also followed closely behind the MP group, revealing the excellent capacity of glucose as an organic carbon source to promote cell growth.

The final contents of the central carbon substances in cells from the three groups were compared (Fig. 1c). *C. zofingiensis* cells showed higher lipid accumulation but lower protein content after MP or HP than AP strategy, reflecting different compound synthesis and interconversion trends. The amino acid composition of heterotrophic cells after MP strategy was particularly analyzed (Table S2); the proportions of glutamate, aspartate, valine, arginine, and leucine are considerable. In order to explore the specific carbon behavioral change, a kinetic carbon distribution model was established. The model appropriately fit the experimental data (*R*^2^ > 0.90); its coefficients are listed in Table 1. The values of *Y*_*PX*_ and *Y*_*CX*_ of three groups maintained at a stable level, indicating that preculture strategies have no apparent effect on substance assimilation for protein and carbohydrate synthesis. In the MP group, *D*_*P*_ reached its highest value; the *α*_*C*_ also showed remarkable improvement after HP or MP, revealing the high conversion rates of proteins and carbohydrates. On the other hand, the elevation of *μ*_*L*_ after MP showed a tendency of heterotrophic *C. zofingiensis* cells to accumulate lipids. The result indicated that the effects of MP and HP on cellular carbon behavior are not in full accord; the former promoted the direct conversion from proteins to lipids, while the latter stimulated carbohydrate breakdown for lipid synthesis.

**Table 1:** Coefficients of the model for carbon partitioning

Coefficient	AP	MP	HP
*Y*_*PX*_ (g g^−1^)	0.2534	0.2469	0.2513
*μ*_*P*_ (h^−1^)	0.0601	0.0875	0.0720
*D*_*P*_ (× 10^−4^ h^−1^)	1.294	2.061	1.734
*Y*_*CX*_ (g g^−1^)	0.3607	0.3611	0.3675
*μ*_*C*_ (h^−1^)	0.0292	0.0330	0.0298
*α*_*C*_ (h^−1^)	0.0045	0.0051	0.0076
*μ*_*L*_ (h^−1^)	0.0155	0.0237	0.0204
*μ* (h^−1^)	0.0165	0.0270	0.0218

### Effect of preculture on the accumulation of value-added compounds

**Fig. 2:**
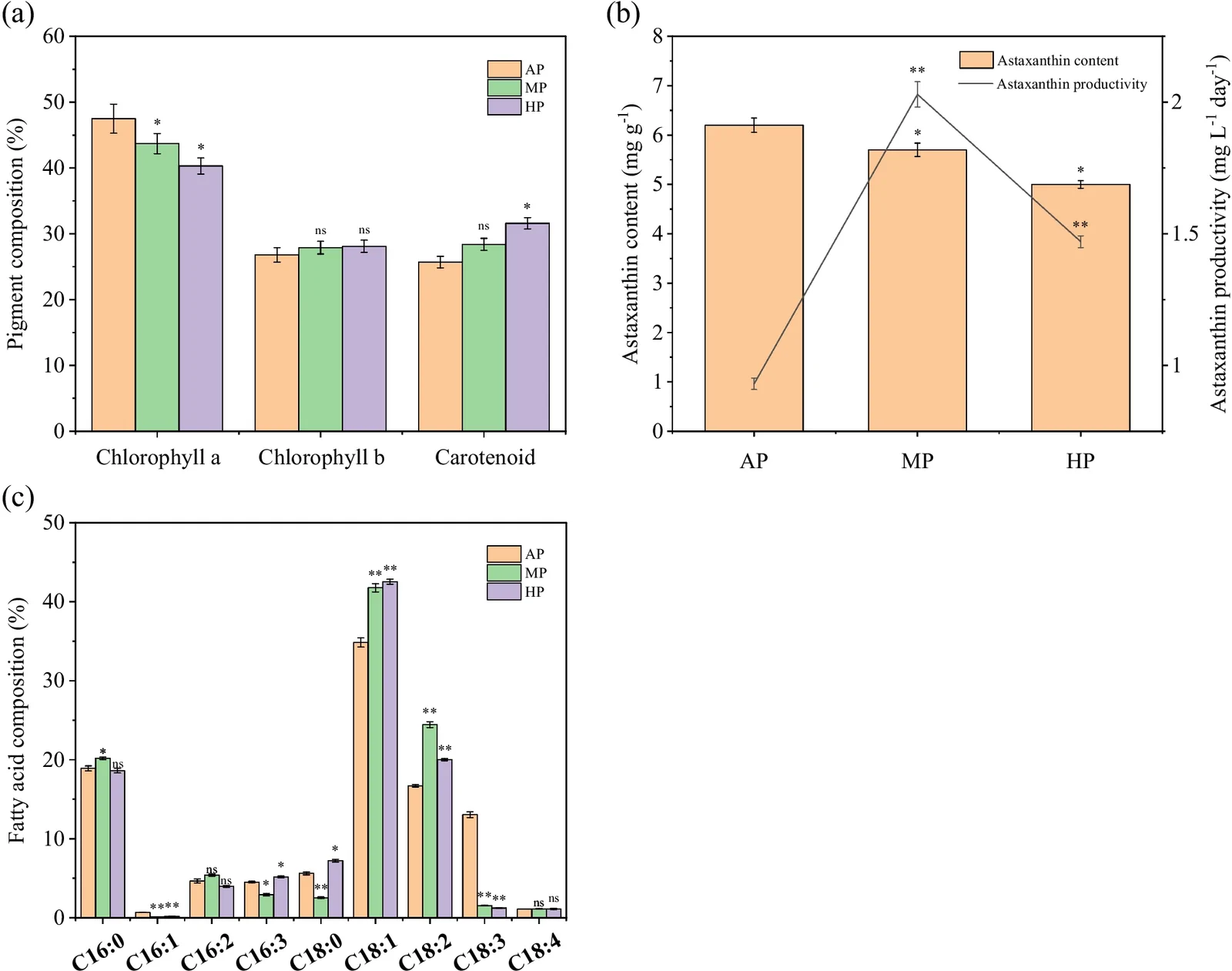
Effect of preculture strategies on pigment composition (**a**), astaxanthin content and productivity (**b**), and fatty acid composition (**c**). Note: the value bar with ns means the difference between the corresponding group and the AP group is not significant, and asterisk denotes statistically significant differences; * *p* < 0.05, ** *p* < 0.01

In *C. zofingiensis*, preculture strategies also influenced the synthesis of significant compounds; Fig. 2 shows their diverse impacts on pigment composition, astaxanthin content and productivity, and fatty acid composition. The chlorophyll a (Chl a) content decreased from 47.5 in AP to 43.7% in MP and 40.3% in HP; during the same period, the contents of Chl b and carotenoids exhibited increasing trends accordingly. The alteration of pigment composition might signify inhibited photosynthesis after MP or HP.

As shown in Fig. 2b, the final astaxanthin contents after AP were 6.20 mg g^−1^, but slightly decreased to 5.70 mg g^−1^ after MP and even declined to 5.00 mg g^−1^ after HP. However, it is essential to consider that ultimate productivity is not solely determined by intracellular astaxanthin content. Biomass concentration also plays a crucial role; the overall productivity can still be improved through accumulating biomass concentration. The final astaxanthin productivity after HP and MP reached 1.47 mg L^−1^ day^−1^ and 2.03 mg L^−1^ day^−1^, which were, respectively, 118% and 58.0% higher than that of the AP group (0.93 mg L^−1^ day^−1^). The results suggested MP and HP as promising approaches for scaled-up production of astaxanthin.

In most astaxanthin-producing organisms, astaxanthin synthesis and accumulation are always tightly linked to lipid metabolism (Patel et al. 2022). As total lipid content got increased, *C. zofingiensis* in the MP and HP groups also displayed higher contents of some specific fatty acids (FAs) than that in the AP group. Generally, FAs in *C. zofingiensis* cells with the same chain length tended to have lower unsaturation after MP or HP (Fig. 2c). Compared with the AP group, the proportion of C16:0, C18:1, and C18:2 in total FA content, respectively, increased by 1.29%, 6.94%, and 7.75% after MP. On the contrary, the proportions of C16:3 and C18:3 decreased by 1.64% and 11.51%, respectively. This desaturation tendency of FAs might be explained by increased respiration and stimulated ROS (reactive oxygen species) formation. Moreover, after MP or HP, the elevated proportions of C18:1 and C16:0 provided sufficient material basis for astaxanthin esterification.

### Transcriptional impact of central carbon metabolism

**Fig. 3:**
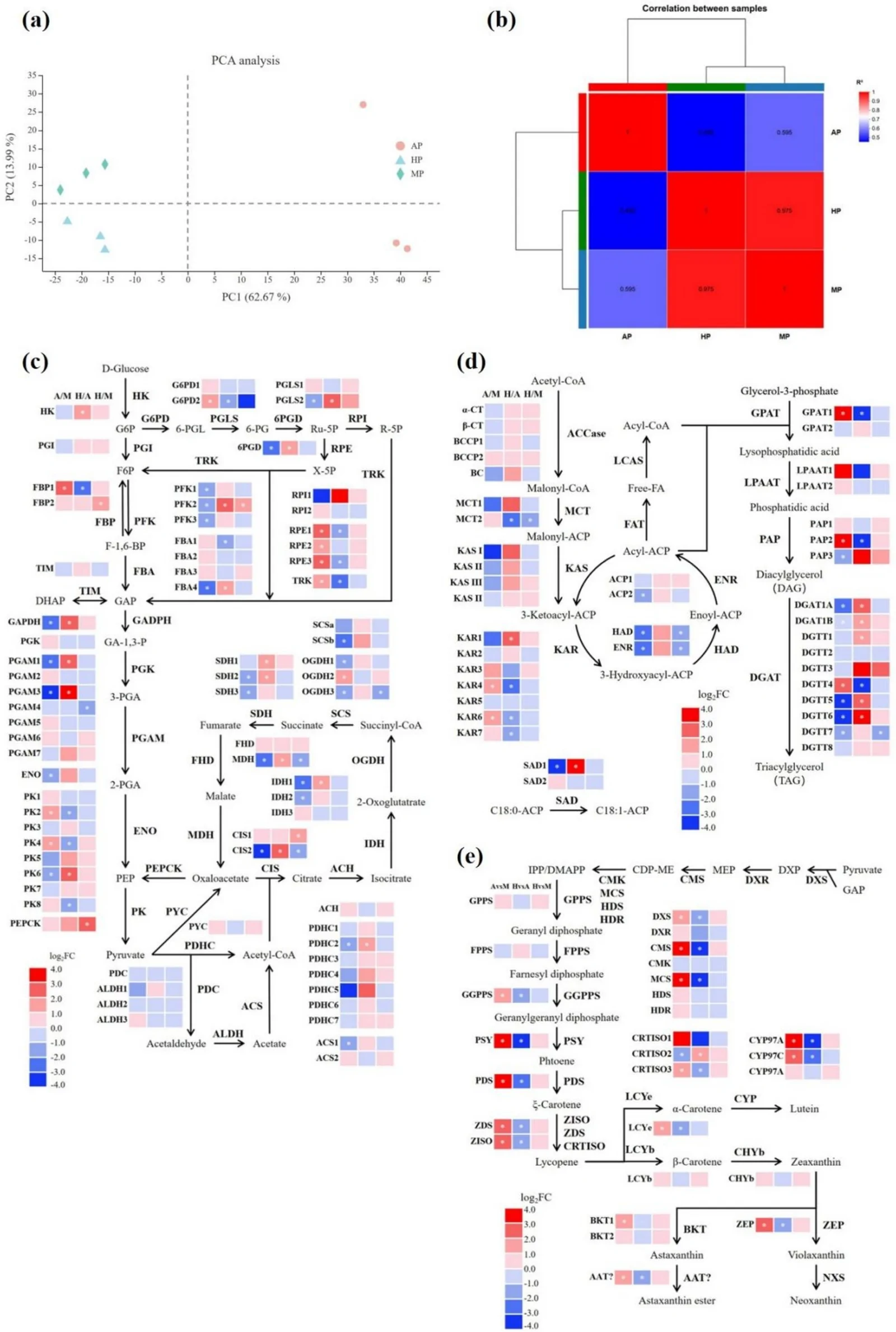
Pearson correlation analysis (**a**) and principal component analysis (PCA) analysis (**b**) of all gene expression levels. Transcriptional regulation of central carbon metabolism (**c**), FA and TAG biosynthesis (**d**), and carotenoids biosynthesis (**e**) in response to different preculture strategies. The asterisk indicates adjusted *p* value (*Padj*) ≤ 0.05. Abbreviations: HK, hexokinase; G6P, glucose-6-phosphate; G6PD, glucose-6-phosphate 1-dehydrogenase; 6-PGL, 6-phosphogluconolactone; PGLS, 6-phosphogluconolactonase; 6-PG, 6-phosphogluconate; 6PGD, 6-phosphogluconate dehydrogenase; Ru-5P, ribulose-5-phosphate; RPI, ribose 5-phosphate isomerase; R-5P, ribose 5-phosphate; RPE, ribulose-phosphate 3-epimerase; X-5P, xylulose 5-phosphate; TRK, transketolase; PGI, glucose-6-phosphate isomerase; F6P, fructose-6-phosphate; FBP, fructose-1,6-bisphosphatase; PFK, phosphofructokinase; F-1,6-BP, fructose-1,6-bisphosphate; FBA, fructose-bisphosphate aldolase; GAP, glyceraldehyde 3-phosphate; TIM, triosephosphate isomerase; DHAP, dihydroxyacetone phosphate; GAPDH, glyceraldehyde 3-phosphate dehydrogenase; GA-1,3-P, glyceraldehyde 1,3-biphosphate; PGK, phosphoglycerate kinase; 3-PGA, glycerol-3-phosphate; PGAM, phosphoglycerate mutase; 2-PGA, glycerol-2-phosphate; ENO, enolase; PEP, phosphoenolpyruvate; PK, pyruvate kinase; PYC, pyruvate carboxylase; CIS, citrate synthase; ACH, aconitate hydratase; IDH, isocitrate dehydrogenase; OGDH, 2-oxoglutarate dehydrogenase; SCS, succinyl-CoA synthetase; SDH, succinate dehydrogenase; FHD, fumarate hydratase; MDH, malate dehydrogenase; PEPCK, phosphoenolpyruvate carboxykinase; PDHC, pyruvate dehydrogenase complex; PDC, pyruvate decarboxylase; ALDH, aldehyde dehydrogenase; ACS, AcCoA synthetase; ACCase, AcCoA carboxylase; CT, carboxyltransferase subunit; BCCP, biotin carboxyl carrier protein; BC, biotin carboxylase; MCT, malonyl-CoA:Acyl carrier protein transacylase; KAS, 3-oxoacyl-[acyl-carrier-protein] synthase; KAR, 3-oxoacyl-[acyl-carrier protein] reductase; HAD, 3-hydroxyacyl-[acyl-carrier-protein] dehydratase; ENR, enoyl-[acyl-carrier protein] reductase; FAT, acyl carrier protein thioesterase; LCAS, long-chain AcCoA synthetase; GPAT, glycerol-3-phosphate acyltransferase; LPAAT, 1-acyl-sn-glycerol-3-phosphate acyltransferase; PAP, phosphatidate phosphatase; DGAT, diacylglycerol *O*-acyltransferase, type I; DGTT, diacylglycerol *O*-acyltransferase, type II; SAD, acyl-[acyl-carrier-protein] desaturase; DXS, 1-deoxy-D-xylulose 5-phosphate synthase; DXP, 1-deoxy-D-xylulose 5-phosphate; DXR, 1-deoxy-D-xylulose 5-phosphate reductoisomerase; MEP, 2-*C*-methyl-D-erythritol 4-phosphate; CMS, 2-*C*-methyl-D-erythritol 4-phosphate cytidylyltransferase; CDP-ME, 4-(cytidine 5-diphosho)-2-*C*-methyl-D-erythritol; CMK, 4-diphosphocytidyl-2-*C*-methyl-D-erythritol kinase; MCS, 2-*C*-methyl-D-erythritol 2,4-cyclodiphosphate synthase; HDS, 4-hydroxy-3-methylbut-2-en-1-yl diphosphate synthase; HDR, 4-hydroxy-3-methylbut-2-enyl diphosphate reductase; IPP, isopentenyl pyrophosphate; DMAPP, dimethylallyl pyrophosphate; GPPS, geranyl diphosphate synthase; FPPS, farnesyl diphosphate synthase; GGPPS, geranylgeranyl diphosphate synthase; PSY, phytoene synthase; PDS, phytoene desaturase; ZISO, zeta-carotene isomerase; ZDS, zeta-carotene desaturase; CRTISO, carotenoid isomerase; LCYe, lycopene epsilon cyclase; LCYb, lycopene beta cyclase; CYP, cytochrome P450 beta hydroxylase; CHYb, beta-carotene hydroxylase; BKT, beta-carotene ketolase; AAT, long-chain-alcohol *O*-fatty-acyltransferase; ZEP, zeaxanthin epoxidase; NXS, neoxanthin synthase

The transcriptome was analyzed to illuminate the behaviors of crucial enzymes in cell metabolism and astaxanthin synthesis after different preculture patterns. Total RNA was extracted for three biological replicates from precultured cells in the three groups. A total of 14,785 genes were detected. Pearson correlation coefficient and PCA analysis reflected high reproducibility among biological replicates (Fig. 3a, b). The changes in transcript abundance between groups were expressed as log2FoldChange (FC), and genes with log2FC ≥ 1 or ≤ − 1 and *P*_*adj*_ ≤ 0.05 were regarded as DEGs. A total of 5782 and 5347 genes were identified as DEGs in AP/MP and HP/AP samples, respectively (Fig. S1). To elucidate the key genes associated with the cellular responses to preculture strategy, DEGs of AP/MP (AP compared with MP) were analyzed using the reference database “KEGG” for pathway classification and functional enrichment (Fig. S2). As seen, DEGs of AP/MP were highly enriched in “photosynthesis-antenna proteins.” The substantially up-regulated DEGs in the “photosynthesis-antenna proteins” category of AP (compared with MP) (Fig. S3) is consistent with the previously observed MP-mediated content reduction of protein and Chl a. The “arginine and proline metabolism” category also contained enriched DEGs (Fig. S2), and the amino acid composition of *C. zofingiensis* after MP is listed in Table S1.

The genes encoding crucial enzymes of the central carbon metabolism and their expression behaviors were delved (Table S1), and the difference in their expression was reduced to a heat map (Fig. 3c). In the pentose phosphate (PP) pathway, the expression levels of genes coding glucose-6-phosphate 1-dehydrogenase (G6PD2), 6-phosphogluconolactonase (PGLS2), and 6-phosphogluconate dehydrogenase (6PGD) significantly increased after MP or HP, compared with AP. Their overexpression can offer sufficient reducing power for lipid and astaxanthin synthesis in *C. zofingiensis*.

Genes involved in glycolysis and gluconeogenesis also got promoted expression after MP or HP. Enolase (ENO) and pyruvate kinase (PK) are responsible for pyruvate synthesis; their augmented transcription can result in higher availability of pyruvate, the essential precursor of carotenoid and lipid synthesis. The gene expression difference between the MP and HP groups was not significant, but distinguishingly, the gene codes phosphoenolpyruvate carboxykinase (PEPCK) controlling the anaplerotic reaction from oxaloacetate (OAA) to pyruvate expressed better in the HP group. The pyruvate dehydrogenase complex (PDHC) and acetyl-CoA synthetase (ACS) catalyzed the conversion from pyruvate to acetyl-CoA (Shtaida et al. 2015). Most genes coding PDHC were strongly up-regulated after MP or HP; ACS-related genes also got improved expression after MP. Therefore, the positive impact of organic carbon source on central carbon metabolism and the synthesis of essential intermediate metabolites has been strongly proven.

The TCA (tricarboxylic acid) cycle is another essential metabolic pathway in *C. zofingiensis*. The expression of related genes was generally elevated after MP or HP, including those coding citrate synthase (CIS2), isocitrate dehydrogenase (IDH1, IDH2), 2-oxoglutarate dehydrogenase (OGDH1, OGDH3), succinyl-CoA synthetase (SCS), succinate dehydrogenase (SDH), and malate dehydrogenase (MDH), indicating that TCA cycle provided more energy to support the substance synthesis under this mode.

### Transcriptional impact of TAG biosynthesis

As shown in Fig. 3d and Table S1, the preculture strategy also impacted the synthesis of triacylglycerol (TAG). In the de novo synthesis pathway of FAs, the regulation effect of gene expression was heterogeneous. Most genes related to 3-oxoacyl-[acyl-carrier protein] reductase (KAR) were up-regulated after AP, but the MP group showed the highest transcription level of 3-hydroxyacyl-ACP-dehydratase (HAD) and enoyl-ACP reductase (ENR). MP and HP displayed similar positive impacts on stearoyl-ACP desaturase (SAD) expression. As to the Kennedy pathway, the involved genes exhibited vastly different expressing behaviors in different groups (Fig. 3d). AP significantly improved the transcription of glycerol-3-phosphate acyltransferase (GPAT1, *Cz11g03260*), 1-acyl-*sn*-glycerol-3-phosphate acyltransferase (LPAAT), and phosphatidate phosphatase (PAP2, *Cz10g16040*); MP and HP tended to increase the expression of genes coding PAP3 (*Cz16g11240*) and five of diacylglycerol acyltransferases (DGAT1A, DGAT1B, DGTT5, DGTT6, and DGTT7). Considering the rate-limiting effect of PAP and DGAT in the Kennedy pathway, the overall TAG biosynthesis has undoubtedly been stimulated in the MP and HP groups.

### Transcriptional impact of astaxanthin biosynthesis

Although the additional organic carbon source in preculture has been identified to improve the production efficiency of astaxanthin, this research still discussed the reason for the decreased intracellular astaxanthin content after MP or HP in depth. In *C. zofingiensis*, all the genes involved in the 2-*C*-methylerythritol 4-phosphate (MEP) and astaxanthin synthesis pathways were identified (Table S1) (Roth et al. 2017). And Fig. S2 displays that the “Carotenoid biosynthesis” category contained enriched DEGs of AP/MP. As predicted, the total transcription of the related enzymes was suppressed after MP or HP (Fig. 3e). Genes coding 1-deoxy-D-xylulose 5-phosphate synthase (DXS), 2-*C*-methyl-D-erythritol 4-phosphate cytidylyltransferase (CMS), and 2-*C*-methyl-D-erythritol 2,4-cyclodiphosphate synthase (MCS) expressed much better after AP, guaranteeing the synthesis activities of isopentenyl pyrophosphate (IPP) and dimethylallyl pyrophosphate (DMAPP).

Owing to sufficient light, the transcriptions of most enzymes in carotenoid synthesis were elevated after AP, including geranylgeranyl diphosphate synthase (GGPPS), phytoene synthase (PSY), phytoene desaturase (PDS), zeta-carotene desaturase (ZDS), zeta-carotene isomerase (ZISO), and carotenoid isomerase (CRTISO). This tendency offered sufficient terpenoid backbones of lycopene, the primary material for astaxanthin biosynthesis. The transcription levels of lycopene beta cyclase (LCYb), beta-carotene hydroxylase (BKT1), and most identified genes in carotenoid synthesis decreased in an order from AP, MP, to HP (Fig. 3e). The declines of transcript abundance of lycopene epsilon cyclase (LCYe), cytochrome P450 beta-hydroxylase (CYP), and BKT1 were dramatic, indicating the restriction of final conversion of terpenoid backbone toward astaxanthin biosynthesis.

Transcriptome analysis revealed that the AP group displayed greater expression levels of genes involved in astaxanthin synthesis, which can be reckoned as the photooxidative stress effect of *C. zofingiensis* for self-protection under lighting condition. On the other hand, both MP and HP displayed the capacity to improve central carbon metabolism and support biomass accumulation, therefore offsetting the backward productivity caused by insufficient astaxanthin content. From the angle of industrial production, MP and HP are undoubtedly better choices. Besides, the increased lipid contents in these two groups might resulted from the enhancing transcription of critical rate-limiting enzymes in the Kennedy pathway.

### Effect of preculture on metabolic flux alteration

**Fig. 4:**
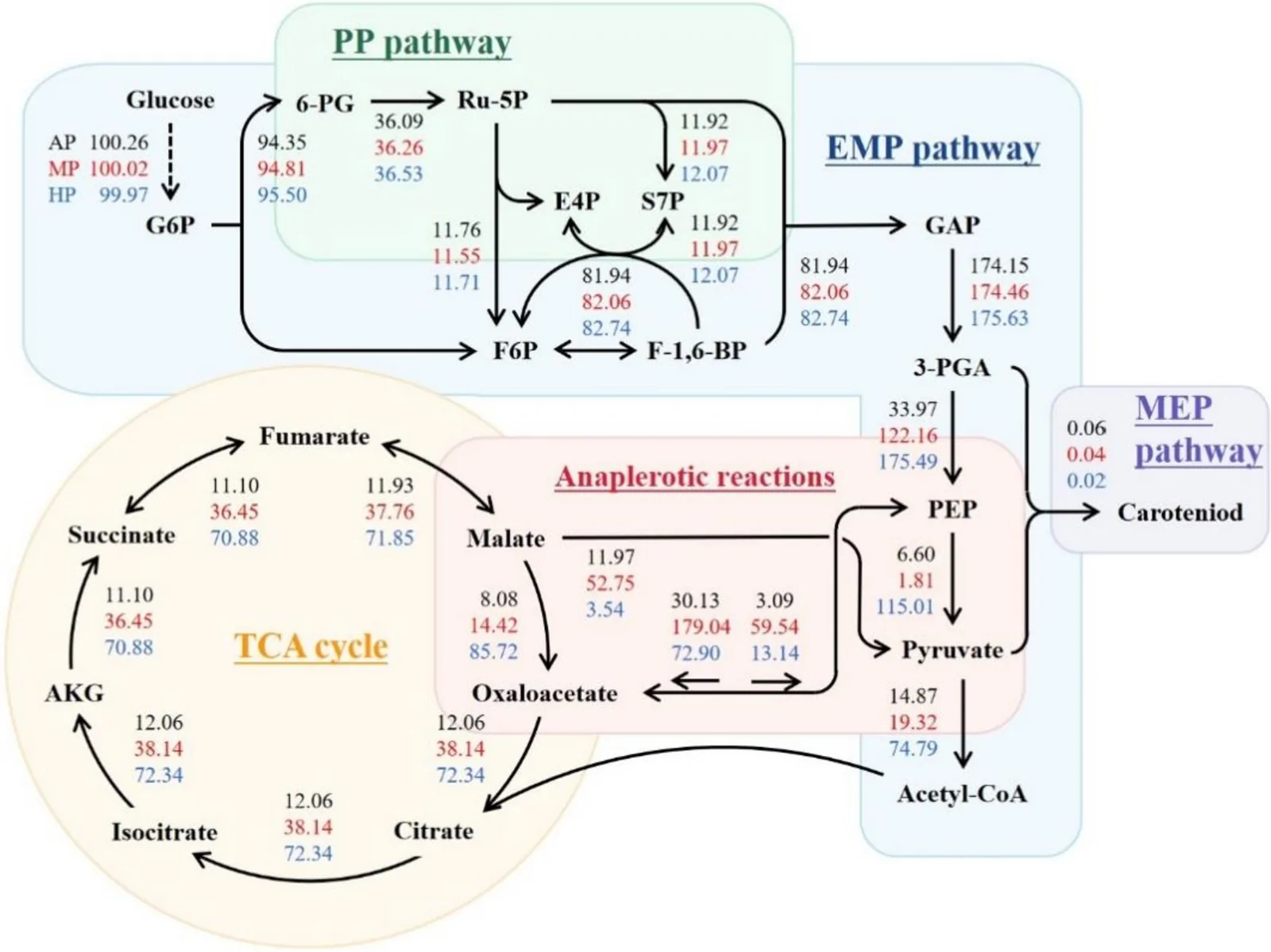
Central carbon flux under different preculture strategies. The flux values of the best fit were normalized to the specific glucose consumption rate of 100. Abbreviations: G6P, glucose-6-phosphate; 6-PG, 6-phosphogluconate; Ru-5P, ribulose-5-phosphate; F6P, fructose-6-phosphate; E4P, erythrose-4-phosphate; S7P, sedo heptulose-7-phosphate; F-1,6-BP, fructose-1,6-bisphosphate; GAP, glyceraldehyde 3-phosphate; 3-PGA, glycerol-3-phosphate; PEP, phosphoenolpyruvate; AKG, α-ketoglutarate

^13^C-MFA was exploited to explore the metabolic pathway rate of *C. zofingiensis* cells under different preculture strategies (Fig. 4). The flux of the PP pathway showed no significant difference under the three strategies, whereas the rates of most reactions in the Embden-Meyerhof pathway (EMP) were accelerated in the MP and HP groups. In particular, the conversion of 3-phosphoglycerate (3-PGA) to phosphoenolpyruvate (PEP) was sharply accelerated, the boosted PEP then turned into pyruvate after HP, but was mainly converted to OAA after MP, namely, the anaplerotic reactions. Via different routes, MP and HP strategies successfully elevated the substance input of TCA cycle and integrally enhanced its activity.

While the cells in the HP group adapted to glucose metabolism more completely, HP also sorely restricted photosynthesis and significantly blocked the carbon flux toward carotenoid synthesis (Fig. 4). In comparison, *C. zofingiensis* cells in the MP group partly retained the carbon flux flowing into carotenoid synthesis, guaranteeing the astaxanthin synthesis ability into some extent.

Overall, HP and MP channeled carbon flux flowing rapidly into the TCA cycle, which supported a faster growth rate of *C. zofingiensis* and led to higher biomass yield. In comparison, MP behaved better at maintaining the carbon flow toward carotenoid synthesis, which explained the higher final astaxanthin content in the MP group.

### Effect of preculture on biomass and astaxanthin productivity in fed-batch culture

**Fig. 5:**
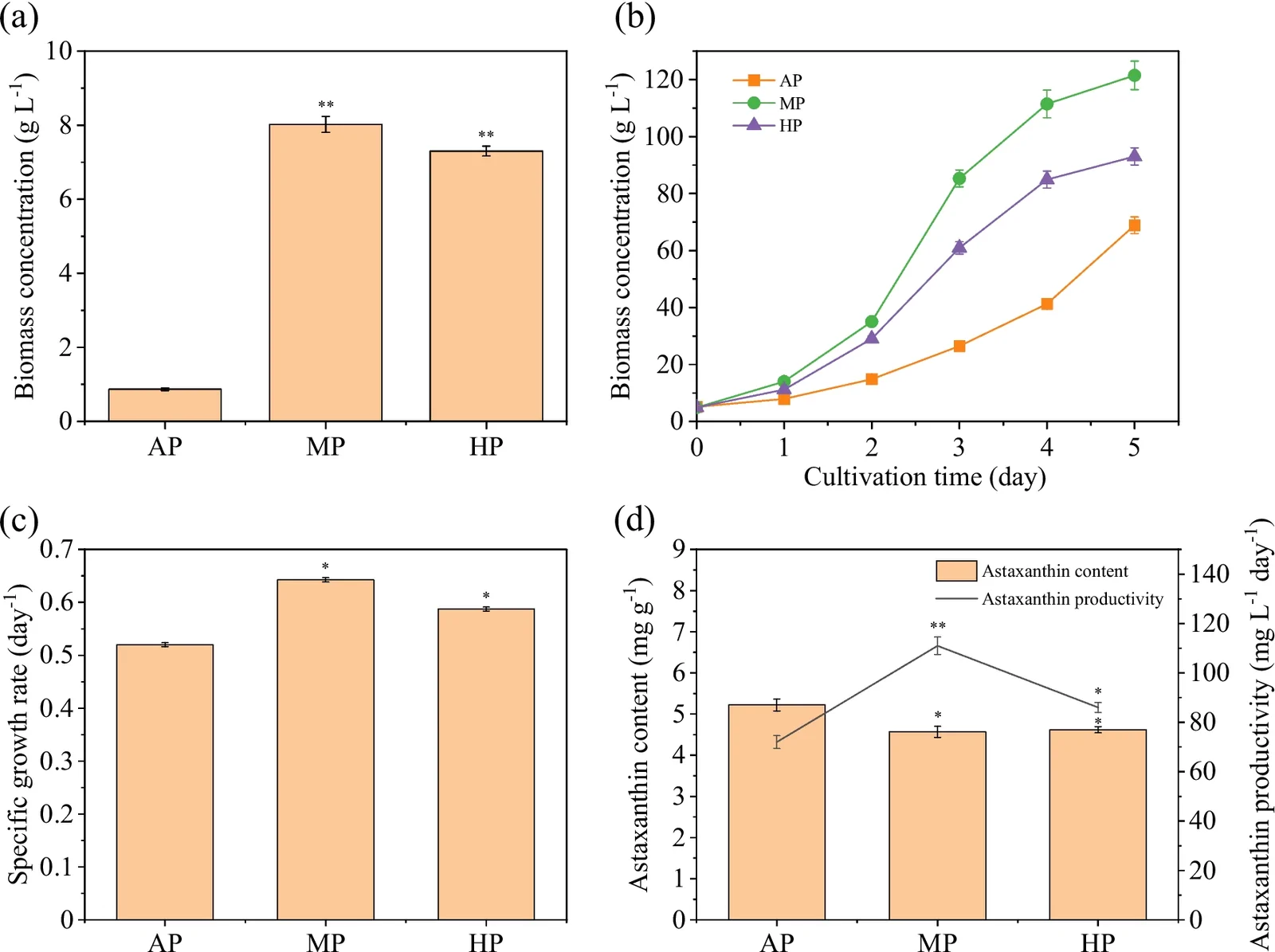
Final biomass concentration of *C. zofingiensis* after FBC in Erlenmeyer flasks (**a**). Final biomass concentration (**b**), specific growth rate (**c**), and astaxanthin content and productivity (**d**) of *C. zofingiensis* after FBC in 20 L fermenters. Note: the value bar with ns means the difference between the corresponding group and the AP group is not significant, and asterisk denotes statistically significant differences; * *p* < 0.05, ** *p* < 0.01

To verify the effect of preculture strategies, an exponential FBC model was designed for cells in heterotrophic period. Initial conditions were set as follows: *X*_0_ = 0.45 g L^−1^, *V*_0_ = 110 mL (in 250 mL Erlenmeyer flasks), *C*_*FG*_ = 400 g L^−1^, *C*_*FN*_ = 97.6 g L^−1^, and *C*_*FP*_ = 13.1 g L^−1^. As obtained in a previous study, *Y*_*XS*_ = 0.5984 g g^−1^ and *μ* = 0.03 h^−1^ (Sun et al. 2021a). Therefore, Eq. (1) can be simplified as *V*_*FS*_ = 0.006204 exp (0.03*t*). Under this condition, *C. zofingiensis* cells maintained the highest specific growth rate to maximize biomass production. By utilizing this FBC model, the biomass concentrations in the AP, MP, and HP groups, respectively, increased by 16%, 351%, and 400% compared to batch culture condition (Fig. 5a). The study further revealed that the MP group demonstrated the greatest ability to promote cell growth. When combined with the FBC model, the MP group achieved a remarkable biomass concentration of 8.02 g L^−1^, nearly 10 times as high as that of the AP group (0.87 g L^−1^). Laboratory-scale fermentation confirmed that the MP strategy holds great promise for the industrial FBC of *C. zofingiensis*.

Finally, the FBC was further developed and implemented in 20 L fermenters. As the culture volume expanded, a new model, *V*_*FS*_ = 0.6204 exp (0.03*t*), was utilized. After MP and 5 days of FBC fermentation, the biomass concentration successfully reached an impressive 121.5 g L^−1^ (Fig. 5b). The specific growth rate of the cells during the exponential growth phase was observed to be as high as 0.64 h^−1^ (Fig. 5c). The stimulated growth rate and increased biomass yield observed with the MP strategy have undoubtedly confirmed its superiority.

As shown in Fig. 5d, although the MP cells exhibited a decreasing trend in astaxanthin content similar to that observed in batch culture, the final astaxanthin productivity reached 111 mg L^−1^ day^−1^, attributing to the significant biomass accumulation. In contrast, the astaxanthin productivity after using the AP and HP strategies was significantly lower, at only 72 and 86 mg L^−1^ day^−1^, respectively. The results strongly proved the superiority of MP combined with large-scale FBC, which not only surpassed the other two preculture methods but also got in the world leading level in industrial astaxanthin production.

## Discussion

Microalgae maintain sophisticated self-regulatory mechanisms to survive in different environments. Compared with *H. pluvialis*, the most prominent advantage of *C. zofingiensis* is its ability to grow heterotrophically and its potential to realize higher astaxanthin productivity (Zhang et al. 2020). Before the heterotrophic period, the usage of different preculture strategies in this study altered the uptake pattern of carbon source of *C. zofingiensis*. MP and HP stimulated rapid cell growth from the first preculture generation, then achieved faster growth rate and higher biomass with improved physiological state and altered intracellular biochemical composition in heterotrophic batch culture. As highlighted in other studies (Ip and Chen 2005; Ip et al. 2004; Zhao et al. 2018), the sharp boosting of biomass demonstrated the superiority of glucose as an organic carbon source for promoting cell growth, revealing MP and HP as potential strategies for industrial *C. zofingiensis* biomass production.

The carbon partitioning model revealed the dynamic regulatory effect of preculture strategy on cell physiological state and intracellular carbon storage. Lipid accumulation is encouraged because of its close relationship to astaxanthin synthesis and accumulation; MP displayed the greatest capacity to stimulate the rapid synthesis of lipid and affiliated high-value components from protein and carbohydrate degradation. From the angle of carbon skeleton source proportion for lipid synthesis, the direct MP-induced carbon flux from proteins toward lipids partly replaced the original path flowing along proteins, carbohydrates, and lipids, which was strengthened after HP. By contrast, the MP strategy ensured sufficient utilization of carbon source, avoided unnecessary intermediate metabolic processes and energy loss, endowed *C. zofingiensis* with the capacity to produce lipids and valuable bioproducts efficiently, and might become a competitiveness strategy in the biofuel industry.

The dramatic change of preculture-induced cellular compositions also covered the modification in the composition of crucial products. Chl a is the primary pigment involved in capturing light energy; its reduced content reflected the impaired function of photosynthetic system in MP and HP (Chen and Blankenship 2011). Under this situation, the cells underwent the chlorophyll breakdown process, where Chl a was converted into Chl b and then into various carotenoids. This breakdown helped to protect cells from oxidative stress caused by ROS (Gill and Tuteja 2010). ROS are highly reactive molecules that can accumulate in cells under stress conditions, such as high light intensity, drought, or temperature extremes. ROS can cause cellular damage by oxidizing important biomolecules like proteins, lipids, and DNA. The accumulated carotenoid could block the oxidative damage of ROS to protect *C. zofingiensis* cells, and further provide abundant precursors for astaxanthin biosynthesis. However, the final astaxanthin contents in MP and HP cells have not risen as expected. Since astaxanthin synthesis from carotenoid precursors is closely linked to photosynthesis (Ip et al. 2004), the synthetic activity of astaxanthin was restrained in HP cells deprived of light; on account of insufficient illumination caused by biomass accumulation, cells in the MP group also showed a slightly decreased activity in related reactions. Fortunately, the inflated biomass ensured the significant improvement of astaxanthin production efficiency. From the industrial perspective, preculture strategies with organic carbon sources master the underlying capacity for promoting commercialized production of astaxanthin.

For most microbial cells, when organic carbon sources are utilized for metabolism and growth, their respiration is generally enhanced and ROS level is increased; the latter is known as oxidative stress that leads to severe damage and even apoptosis (Liu et al. 2015). The synthesis pathway of FAs, especially polyunsaturated fatty acids (PUFAs), serves as the electron sinks (Lemoine and Schoefs 2010) and acts as antioxidants to mediate oxidative stress by effectively quenching ROS. Thus, the enhanced ROS production may be an important factor in the consumption of PUFAs, which can be a reasonable explanation for the overall desaturation of FAs (Johansson et al. 2016).

Furthermore, the fatty acid moieties of astaxanthin monoester and diester consisted mainly of C18:1 and C16:0 (Roth et al. 2017). The up-regulation of C18:1 and C16:0 proportions after organic carbon preculture can offer sufficient fatty acid moieties of astaxanthin monoester and diester, guaranteeing the rapid esterification of astaxanthin (Zhang et al. 2020). Since MP and HP also accelerated total lipid synthesis and up-regulated PAP and DGAP transcription level, the astaxanthin esters can be immediately wrapped in TAG-filled lipid droplets to maintain their stability. As to C18:3, its prominent content after AP could be explained as the result of the close relationship between its synthesis and photosynthetic carbon assimilation (Harwood and Guschina 2009).

Besides, as crucial high-value components, microalgal lipids enriched with FAs are also important substances for multiple bioproducts (Ahmad et al. 2022). For instance, C16:0 and C18:1 are preferred for their important biofuel properties. The change of FA composition after MP offered *C. zofingiensis* with unique combustion properties, such as better cold flow properties and oxidative stability, when applied as materials or propellants of biofuel. The saturated fatty acid content gives the biofuel a higher cetane number and enhances its stability. The monounsaturated fatty acids (MUFAs) contribute to improving cold flow properties, while moderate PUFAs help in low-temperature properties and oxidative stability (Guldhe et al. 2019). C18:1 and C18:2, which got sufficient enrichment in MP cells, are crucial intermediate molecules of high-value long-chain FA synthesis such as eicosapentaenoic acid (EPA) and docosahexaenoic acid (DHA). All these changes in FA composition after preculture with glucose, particularly MP strategy, endowed *C. zofingiensis* excellent application value in biofuels, dietary, infant food, medication, animal feeds, and cosmetics, thus broadening its commercial develop domain.

Since intracellular astaxanthin is stored in TAG-filled lipid droplets, the synthesis of TAG is closely connected to astaxanthin synthesis and accumulation. The transcriptional levels of the genes appertained to TAG and carotenoid synthesis were analyzed, and the carbon flux of central metabolism was explored by ^13^C-MFA. As shown in transcriptome results, the de novo FA synthesis pathway, the primary source of acyl substrates, was up-regulated after MP. TAG is synthesized by esterifying FAs in the Kennedy pathway (Mao et al. 2019). While the transcriptional level of GPAT1 and PAP2 decreased, which led to the accumulation of acyl-CoA, the up-regulated expression level of rate-limiting enzymes PAP3 and most of DGAT genes strongly promoted TAG synthesis. The differential transcription of DGAT homologs is linked to functional divergence (Mao et al. 2020).

Despite the augmented pyruvate pool after organic carbon preculture, the expression of most genes in the MEP pathway showed no apparent alteration. The synthesis activities of IPP and DMAPP, the direct precursors for carotenoid synthesis, were significantly suppressed in the MP and HP groups due to the low transcription levels of MEP-related enzymes. Similarly, the activity of enzymes involved in carotenoid synthesis, especially LCYb that redirected lycopene to β-carotene and BKT1 that reallocated zeaxanthin to astaxanthin (Zhang et al. 2020), showed a decline after organic carbon preculture, resulting in restricted terpenoid backbone supply for astaxanthin biosynthesis. This trend is mainly attributed to the tight connection between carotenoid synthesis and photosynthesis (Vilchez et al. 2011). In the presence of organic carbon sources, *C. zofingiensis* got rid of dependence on energy supply from photosynthesis and thus reduced the activity of related reactions. Moreover, transcript abundance of long-chain-alcohol *O*-fatty-acyltransferase (AAT), the enzyme involved in the esterification of astaxanthin in *C. zofingiensis* (Roth et al. 2017), was also substantially reduced after MP or HP, which restricted the stable existence of synthesized astaxanthin in TAG-filled lipid droplets as astaxanthin ester. The lack of carotenoid precursors and decreased enzyme activity jointly resulted in a descend in turn of intracellular astaxanthin after AP, MP, and HP. HP inhibited the carbon flow into carotenoid synthesis to a severe degree and certainly limited the synthesis activity of astaxanthin. By contrast, the carbon flow toward carotenoid synthesis in MP cells was partially preserved, and excellent astaxanthin yield was obtained due to superior biomass concentration.

ROS accumulation from respiration can also explain the gradual decrease of intracellular astaxanthin content among the AP, MP, and HP groups. The enhanced activity of the TCA cycle indicated that *C. zofingiensis* relies more on respiration for energy production and carbon availability augmentation. Similar to other antioxidative carotenoids, astaxanthin has been proven to effectively protect algal cells against oxidative damage by quenching excessive ROS and other free radicals under stress conditions (Vilchez et al. 2011). Therefore, ROS generation can lead to the consumption of astaxanthin and its precursors. On the other hand, due to the sharp amplification of biomass in the MP group, the reduction of effective light intensity caused by the shielding between cells is another reason for the block of astaxanthin accumulation. While *C. zofingiensis* can produce astaxanthin in darkness, it still shows a typical light-dependent growth, and astaxanthin synthesis can still be induced by light intensity, for this process has been identified as a protective response stimulated by photo-oxidative stress (Del Campo et al. 2004).

From the perspective of metabolite synthesis, MP may be the most suitable preculture strategy for large-scale commercial astaxanthin production. This conclusion could be proved by its critical role in regulating crucial carbon metabolic pathways. MP cells mastered the greatest balance between the increased glucose metabolism and the inhibition of carotenoid synthesis. The glycolytic pathway provides pyruvate and glyceraldehyde 3-phosphate (GAP), and the former can convert to essential ingredients of central carbon metabolism. Moreover, as an irreplaceable precursor, pyruvate accessibility has a decisive influence on the production of lipids and carotenoids. ENO catalyzes the conversion of 2-phosphoglycerate (2-PGA) to PEP, and PK directly regulates pyruvate synthesis (McKie-Krisberg et al. 2018). Their augmented transcription after MP can result in higher availability of pyruvate, the essential precursor of carotenoid and lipid synthesis. As the direct downstream of pyruvate, acetyl-CoA is the essential intermediary metabolism substance in cell growth and compound synthesis of microalgae (Sun et al. 2020b). As the initial block of extending the acyl chain, it is the central cellular metabolite of the TCA circle. Moreover, acetyl-CoA is both the precursor for FA and carotenoid synthesis and a product of the degradation pathways of FA and some amino acids; its efficiency and availability are pivotal for FA and carotenoid synthesis (Shtaida et al. 2015). Acetyl-CoA can be generated from pyruvate, catalyzed by PDHC, or be conversed from acetate, in the presence of ACS (Shtaida et al. 2015). The significant up-regulated expression level of PDHC and ACS after MP strengthened the production and availability of cellular acetyl-CoA. Meanwhile, the down-regulated overall expression of genes related to the Kennedy pathway inhibited the transformation of 3-PGA, further aggravating acetyl-CoA retention.

In addition to abundant substrate molecules and enzymes, astaxanthin synthesis also requires NADPH and ATP to provide reducing power and energy (McKie-Krisberg et al. 2018). The PP pathway has long been recognized as the primary source of NADPH and ATP under stress conditions, especially when the photosynthesis in *C. zofingiensis* is hindered (Xue et al. 2018). This pathway generates two NADPH molecules for each G6P molecule catalyzed and is also strongly associated with FA and astaxanthin accumulation. The up-regulated transcription of the related enzymes, especially 6PGD, could catalyze NADPH production and consequently provide sufficient reducing power and energy for intracellular biosynthetic processes, and the increased reductant pool could improve the anabolism (Mao et al. 2020).

The absorbed glucose is generally guided into EMP for providing ATP to support cell growth and bioactivities. The strengthened EMP metabolic flux revealed by ^13^C-MFA reflected the adaptation of *C. zofingiensis* cells to glucose metabolism. The anaplerotic reactions, together with TCA cycle, got enhanced metabolic flux after MP, thus boosting pyruvate accumulation and NADPH production, and channeling carbon flow rapidly into TCA cycle. The importance and necessity of the TCA cycle in cell metabolism has been clarified in numerous organisms; this pathway is the crucial hub in charge of substance supply and energy metabolism for various physiological activities (Prasun 2019). Oxidation of 1 pyruvate molecule through the TCA cycle can yield 15 ATP equivalents (Engelking 2010). The TCA cycle produces 32 or 30 molecules of ATP from each glucose molecule with aerobic oxidation. With glucose absorbed into *C. zofingiensis* cells, HP and MP channeled carbon flow flowing rapidly into the TCA cycle, strengthened the TCA cycle and respiration, and provided *C. zofingiensis* with faster growth rates and higher biomass yield. For the HP group, this tendency seemed unsurprising because it is widely known that *C. zofingiensis* relies on respiration under the restriction of photosynthesis. As mentioned, HP sorely restricted related-gene expression and carbon distribution for carotenoid synthesis, which led to a considerable decline in astaxanthin content. In comparison, the MP group was possibly adapted to organic carbon source and began to rely on the TCA cycle for material and energy supply to some extent, leading to much higher biomass yield than the AP group. Meanwhile, PYR content can be generated through reversible anaplerotic reactions from malate (MAL) after MP. Anaplerotic reactions are the intracellular process that produces TCA intermediates such as OAA and MAL from PYR or PEP, thus balancing the rates of the EMP pathway, the PP pathway, and the TCA cycle (Latorre-Muro et al. 2018). The increased fractional percentage of PYR from MAL improved PYR availability and NADPH synthesis activity after MP and guaranteed a better precursor content for carotenoid synthesis than the HP group. In a word, MP successfully endowed the precultured cells with a relative balance of metabolism state, by which cell growth and division could be significantly accelerated while avoiding the severe inhibition of carotenoid synthesis. Thus, the MP strategy elevated the total productivity of astaxanthin from two aspects: accumulated large amounts of biomass and prevention from severely reducing of intracellular astaxanthin content, displaying its commercial advantage over AP and HP.

Modeling growth is effective for studying and adjusting the growth performance of microorganisms; it helps control microalgae growth in artificial bioreactors (Yuan et al. 2014). Exponential fed-batch culture can maximize the growth rate of microalgae by maintaining nutrient content of the system at the most suitable level. This culture pattern has been proven effective in promoting biomass accumulation and increasing cell density (Brignoli et al. 2020). When combined with the FBC model, *C. zofingiensis* after MP could further demonstrate more competitive specific growth rate and resulted in a further amplified biomass yield. MP strategy also provided sufficient material for acquiring of astaxanthin and endowed *C. zofingiensis* with a greater value for industrialization. While the astaxanthin content of MP cells showed a decreasing trend, the final astaxanthin productivity achieved 111 mg L^−1^ day^−1^, ascribed to the vast biomass accumulation. This yield was more than 7-fold higher than the previously recorded highest productivities of *C. zofingiensis* (Liu et al. 2013) and *H. pluvialis* (Lu et al. 2019). Combining the MP strategy and the FBC model boosted biomass yield and resulted in a substantial leap in astaxanthin productivity, signifying a significant step forward to commercializing natural astaxanthin.

While AP displayed the best capacity to stimulate intracellular astaxanthin synthesis, MP combined with FBC successfully elevated the astaxanthin productivity of the fermentation system, owing to the excellent biomass accumulation. Regarding economical industrial production, MP is undoubtedly the most appropriate preculture strategy for enhancing producing efficiency and meanwhile reducing production costs. The fermentation of *C. zofingiensis* consists of various essential steps such as culture media preparation, microalgae growth, biomass harvesting, bioreactor cleaning, and so on (Sui et al. 2020). With the development of numerous new methods or devices, the average production cost of commercial astaxanthin fermented by microalgae is now around 552 USD kg^−1^, and its selling price can reach over 2500 USD kg^−1^ (Lafarga and Acien 2021).

MP mainly contributes to reduce the equipment and operating costs in the fermentation stage, which accounts for nearly 45% of the total cost (Ruiz et al. 2016). Compared with traditional methods, fermentation strategy with MP requires 4-fold lower land cost, 50-fold lower fresh water cost, 8-fold lower equipment cost, and 4-fold shorter formal fermentation time (as the reported shortest period is 21 days) (Chen et al. 2022; Janssen et al. 2022). The additional cost of the MP strategy focuses on utilizing glucose and illumination in the preculture stage. Since the preculture system is small (only one-tenth of the heterotrophic fermentation system), these two parts, respectively, require additional costs of 5.4 and 9.7 USD kg^−1^ (Janssen et al. 2022; Wang et al. 2023a). In contrast, AP is independent of glucose input, so the material cost of the AP strategy is estimated to be around 11.7 USD kg^−1^. However, MP successfully increases the production efficiency of a single fermentation system up to seven times, which is difficult to achieve in the other two strategies. Based on previous data on the cost of each part, MP may greatly reduce the production costs by 25%, and control the astaxanthin production cost of *C. zofingiensis* within 500 USD kg^−1^. Therefore, MP shows full economic feasibility and has considerable economic benefits in commercial applications. The output of high-value products from microalgae cultivation has been recognized as an eco-friendly biorefining technology, and could even contribute to global carbon neutrality. Moreover, the MP strategy avoids the usage of any toxic or harmful additives and largely simplifies the production process; when combined with FBC, it can also guarantee the full use of nutrition resources (Wang et al. 2023a). Consequently, MP displays little environmental impact throughout the life cycle of astaxanthin production, which aligns with the modern society’s pursuit of zero-carbon circular bioeconomy.

To sum up, heterotrophic cultivation of *C. zofingiensis* could be the viable approach to industrial astaxanthin production, and this study emphasized the superiority of MP strategy in astaxanthin production. MP enhanced the biomass yield of *C. zofingiensis*, and endows heterotrophic *C. zofingiensis* with application possibility in efficient production of astaxanthin, which deserves deeper development at the industrial level. Besides, promoting biosynthesis of low unsaturated fatty acids, represented by C16:0 and C18:1, facilitated by MP, could ensure the astaxanthin stability and endow *C. zofingiensis* with application value in healthcare and biofuel fields. This strategy may finally contribute to the industrialization and commercialization of natural astaxanthin and other high-value microalgal metabolites.

## Supplementary information

ESM 1 (PDF 897 kb)

## Data Availability

Data will be made available on request.
