# Supplementary material for: Selecting a preculture strategy for improving biomass and astaxanthin productivity of Chromochloris zofingiensis
Source: Appl Microbiol Biotechnol. 2024 Jan 10;108:117. doi: 10.1007/s00253-023-12873-x (PMC10781847; doi:10.1007/s00253-023-12873-x)
Supplement: Supplementary file 1 [file 253_2023_12873_MOESM1_ESM.pdf]

Applied Microbiology and Biotechnology

Supplementary Material

Selecting a preculture strategy for improving biomass and astaxanthin productivity of *Chromochloris zofingiensis*

Yuxin Wang [1], Jia Wang [1], Shufang Yang [2], Qingping Liang [1], Ziqiang Gu [1], Ying Wang [3,*], Haijin Mou [1,*], Han Sun [2,*]

[1] College of Food Science and Engineering, Ocean University of China, Qingdao 266003, China;

[2] Institute for Advanced Study, Shenzhen University, Shenzhen 518060, China;

[3] Marine Science research Institute of Shandong Province, Qingdao, 266003, China

[*] Correspondence concerning this article should be addressed to Prof. Ying Wang, Email: food_rc@sina.com; Prof. Haijin Mou, Email: mousun@ouc.edu.cn and Dr. Han Sun, Email: shlyg2242@163.com

Tables

Table S1 Expression patterns of genes involved in TAG, astaxanthin biosynthesis and central carbon mechanism.

Table S2 Amino acid composition of *C. zofingiensis* after MP.

Figures

Fig. S1. Volcano plot of DEGs. (A) AP/MP. (B) HP/MP. (C) HP/AP.

Fig. S2. KEGG pathway classification and enrichment of AP/MP DEGs. (A) KEGG pathway classification of AP/MP. (B) KEGG pathway functional enrichment of AP/MP. Rich factor indicates the ratio of DEGs to all genes in that pathway.

Fig. S3. Transcriptional regulation of "photosynthesis-antenna protein" KEGG pathway. The red and blue boxes indicate up-regulation and down-regulation respectively.

Fig. S1. Volcano plot of DEGs. (A) AP/MP. (B) HP/MP. (C) HP/AP.

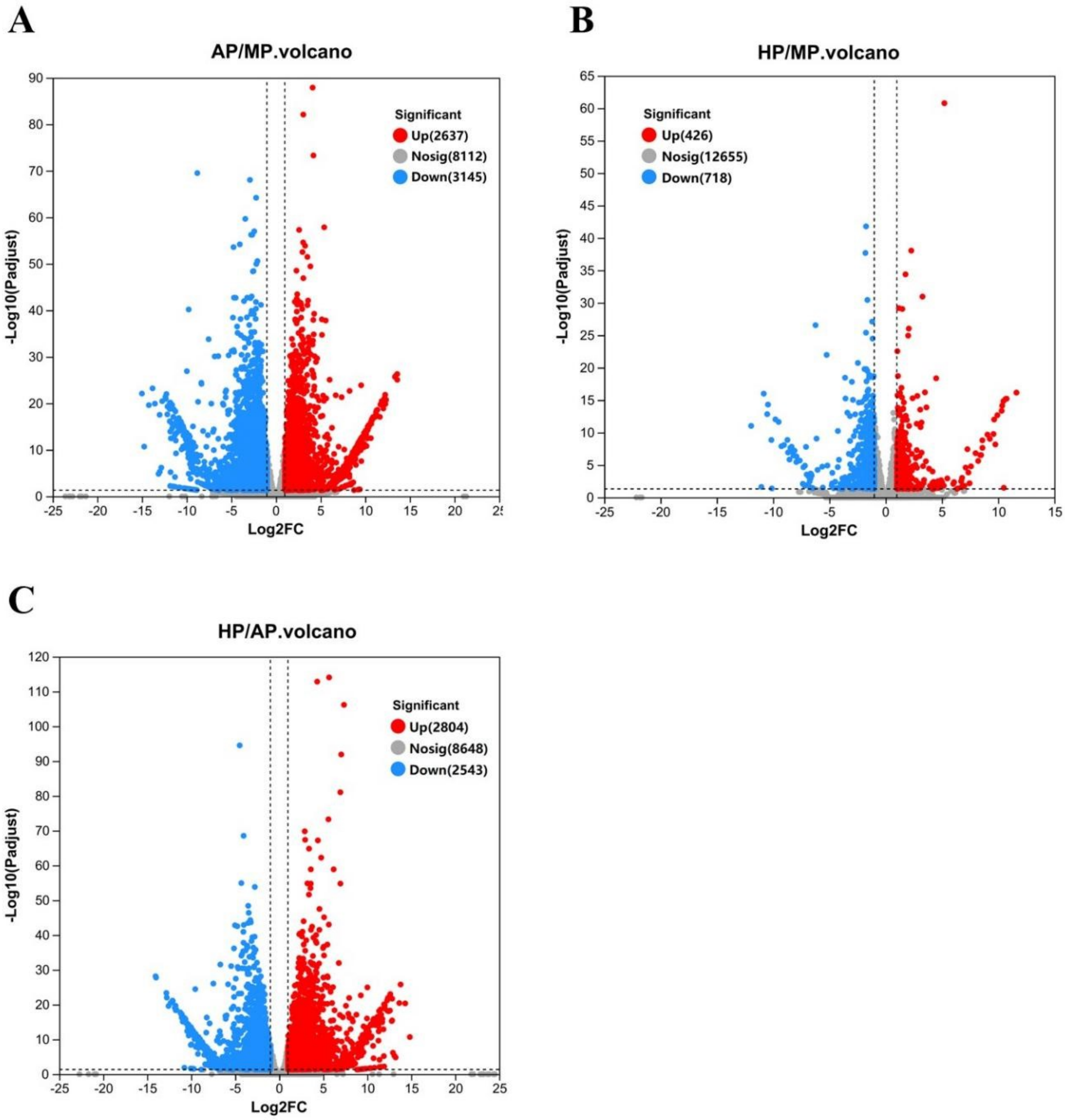

Fig. S2. KEGG pathway classification and enrichment of AP/MP DEGs. (A) KEGG pathway classification of AP/MP. (B) KEGG pathway functional enrichment of AP/MP. Rich factor indicates the ratio of DEGs to all genes in that pathway.

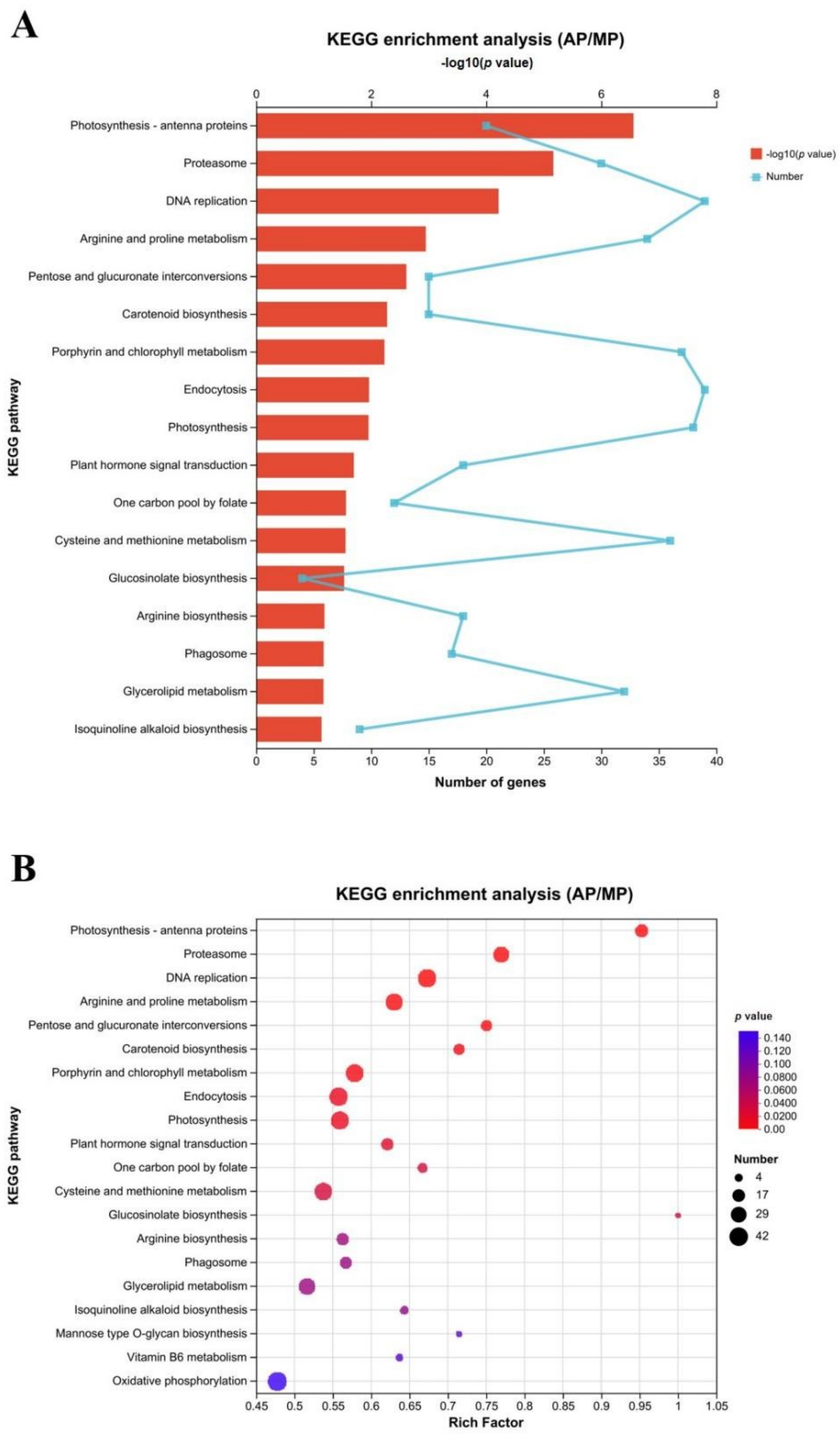

Fig. S3. Transcriptional regulation of of AP/MP KEGG pathway of "photosynthesis-antenna protein". The red and blue boxes indicate up-regulation and down-regulation respectively.

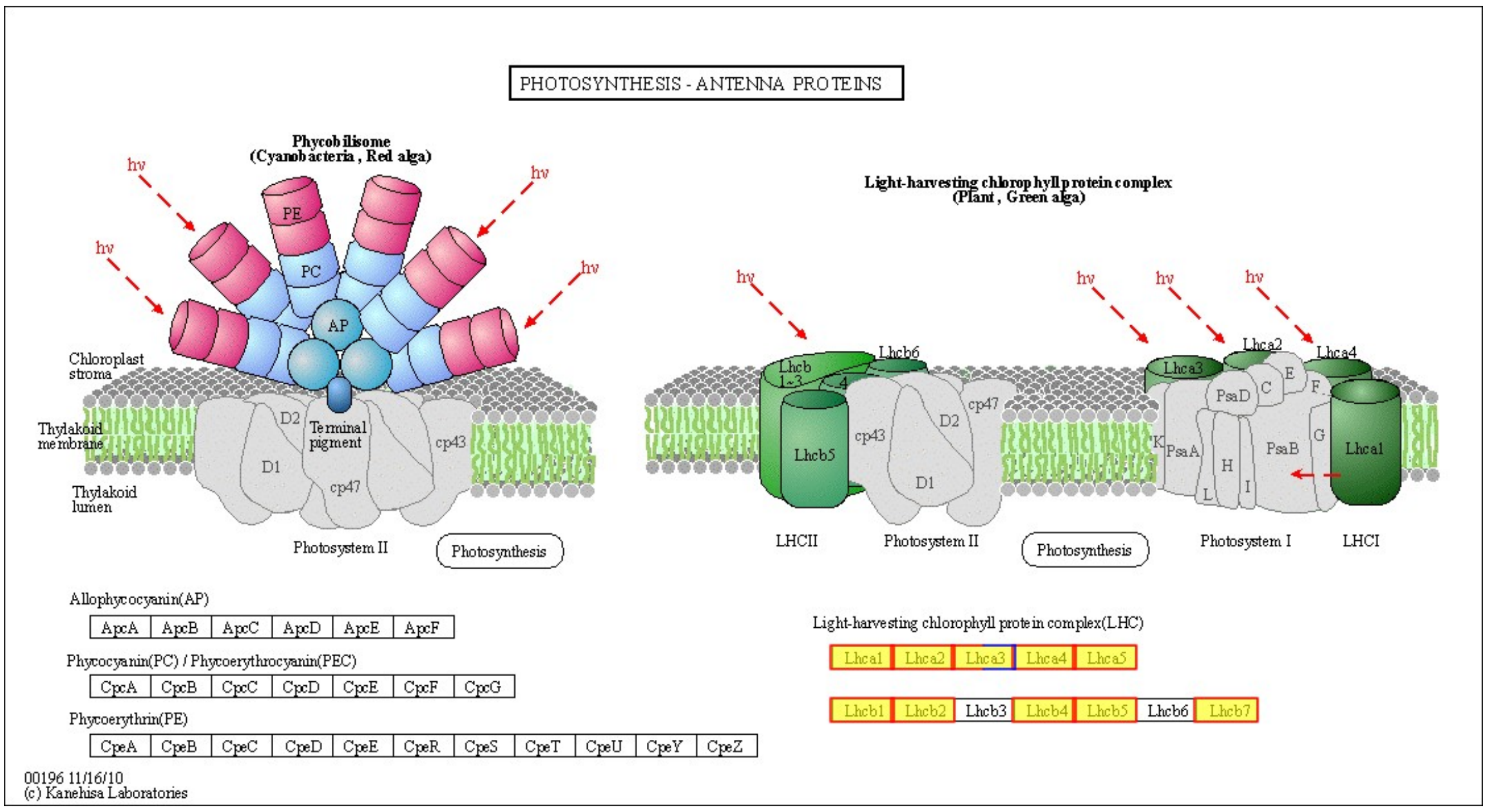

Table S1 Expression patterns of genes involved in TAG, astaxanthin biosynthesis and central carbon mechanism

Gene ID	Annotation	Abbreviation	log 2 FC AP/MP	HP/AP	HP/MP
**FA biosynthesis**					
Cz02g12030	Carboxyltransferase subunit alpha (ACCase complex)	*α-CT*	-0.45	0.73	0.29
Cz02g17060	Carboxyltransferase subunit beta (ACCase complex)	*β-CT*	-0.05	0.55	0.51
Cz03g28270	Biotin carboxyl carrier protein (ACCase complex)	*BCCP1*	-0.83	0.80	-0.02
Cz06g20040	Biotin carboxyl carrier protein (ACCase complex)	*BCCP2*	0.74	0.09	0.83
Cz13g10110	Biotin carboxylase (ACCase complex)	*BC*	-1.53	1.38	-0.13
Cz19g10190	Acetyl-CoA carboxylase	*ACCase*	-0.59	0.79	0.22
Cz09g30220	Acyl carrier protein	*ACP1*	-0.89	0.93	0.05
Cz07g17120	Acyl carrier protein	*ACP2*	-1.11	0.98	-0.11
Cz13g05150	Malonyl-CoA:Acyl carrier protein transacylase	*MCT1*	-2.29	2.10	-0.17
Cz04g37050	Malonyl-CoA:Acyl carrier protein transacylase	*MCT2*	0.99	-2.29	-1.27
UNPLg00257	3-Oxoacyl-[acyl-carrier-protein] synthase, II (C16:0 to C18:0)	*KAS II*	-1.54	1.25	-0.27
Cz02g14160	3-Oxoacyl-[acyl-carrier-protein] synthase, I (C4:0 to up to C16:0)	*KAS I*	-3.17	2.78	-0.37
Cz18g03070	3-Oxoacyl-[acyl-carrier-protein] synthase, III (C2:0 to C4:0)	*KAS III*	-1.00	1.05	0.07
Cz06g14030	3-Oxoacyl-[acyl-carrier-protein] synthase, II	*KAS II*	-0.66	0.69	0.04
Cz01g34370	3-Oxoacyl-[acyl-carrier protein] reductase	*KAR1*	-2.48	2.53	0.06
Cz16g00050	3-Oxoacyl-[acyl-carrier protein] reductase	*KAR2*	-0.64	0.56	-0.06
Cz10g18200	3-Oxoacyl-[acyl-carrier protein] reductase	*KAR3*	1.55	-1.10	0.47
Cz03g39140	3-Oxoacyl-[acyl-carrier protein] reductase?	*KAR4*	1.48	-2.06	-0.57
Cz15g00050	3-Oxoacyl-[acyl-carrier protein] reductase	*KAR5*	-0.43	-0.09	-0.51
Cz04g17270	3-Oxoacyl-[acyl-carrier protein] reductase	*KAR6*	1.82	-1.20	-0.60
Cz11g27250	3-Oxoacyl-[acyl-carrier protein] reductase?	*KAR7*	0.56	-1.33	-0.75
Cz01g09160	3-Hydroxyacyl-[acyl-carrier-protein] dehydratase	*HAD*	-2.87	1.79	-1.06
Cz11g20040	Enoyl-[acyl-carrier protein] reductase	*ENR*	-2.69	1.54	-1.14
**FA desaturation**					
Cz04g09090	Acyl-[acyl-carrier-protein] desaturase	*SAD1*	-4.16	3.35	-0.79
Cz13g17200	Acyl-[acyl-carrier-protein] desaturase	*SAD2*	0.47	-0.92	-0.44

**TAG synthesis**					
Cz11g03260	Glycerol-3-phosphate acyltransferase	*GPAT1*	8.96	-9.90	-0.91
Cz09g31330	Glycerol-3-phosphate acyltransferase	*GPAT2*	-0.97	0.93	-0.02
Cz16g02090	1-Acyl-*sn*-glycerol-3-phosphate acyltransferase	*LPAAT1*	10.00	-10.00	0.02
Cz10g20070	1-Acyl-*sn*-glycerol-3-phosphate acyltransferase	*LPAAT2*	0.17	-0.27	-0.08
Cz05g23060	Phosphatidate phosphatase, Lipin	*PAP1*	0.70	-0.19	0.53
Cz10g16040	Phosphatidate phosphatase	*PAP2*	5.24	-4.97	0.29
Cz16g11240	Phosphatidate phosphatase	*PAP3*	-1.78	3.69	1.91
Cz10g01120	2-Acylglycerol *O*-acyltransferase	*MGAT*	7.13	-7.38	-0.22
Cz06g05010	Diacylglycerol *O*-acyltransferase, type I	*DGAT1A*	-2.15	2.05	-0.08
Cz09g08290	Diacylglycerol *O*-acyltransferase, type I	*DGAT1B*	1.78	-1.53	0.27
Cz06g35060	Diacylglycerol *O*-acyltransferase, type II	*DGTT1*	-0.88	1.76	0.91
Cz06g22030	Diacylglycerol *O*-acyltransferase, type II	*DGTT2*	-0.46	-0.35	-0.78
Cz09g23010	Diacylglycerol *O*-acyltransferase, type II	*DGTT3*	-0.56	3.49	2.95
Cz11g24150	Diacylglycerol *O*-acyltransferase, type II	*DGTT4*	2.73	-3.24	-0.49
Cz09g27290	Diacylglycerol *O*-acyltransferase, type II	*DGTT5*	-3.16	2.56	-0.59
Cz15g22140	Diacylglycerol *O*-acyltransferase, type II	*DGTT6*	-12.26	12.35	0.00
Cz11g21100	Diacylglycerol *O*-acyltransferase, type II	*DGTT7*	-1.89	0.52	-1.35
Cz08g14220	Phospholipid:diacylglycerol acyltransferase	*DGTT8*	-0.01	0.21	0.21
**Carotenoids synthesis**					
Cz02g35280	1-Deoxy-D-xylulose 5-phosphate synthase (DOXP synthase)	*DXS*	1.25	-1.02	0.24
Cz07g19130	1-Deoxy-D-xylulose 5-phosphate reductoisomerase	*DXR*	0.60	-1.01	-0.40
Cz12g10090	2-C-methyl-D-erythritol 4-phosphate cytidylyltransferase	*CMS*	9.12	-8.48	0.67
Cz11g24270	4-Diphosphocytidyl-2-C-methyl-D-erythritol kinase	*CMK*	-0.02	-0.26	-0.27
Cz02g10180	2-C-methyl-D-erythritol 2,4-cyclodiphosphate synthase	*MCS*	5.23	-5.92	-0.68
Cz13g08020	4-Hydroxy-3-methylbut-2-en-1-yl diphosphate synthase	*HDS*	0.36	-0.60	-0.23
Cz05g23010	4-Hydroxy-3-methylbut-2-enyl diphosphate reductase	*HDR*	0.57	-0.88	-0.30
Cz04g25080	Acetoacetyl-CoA thiolase	*AACT*	-0.70	0.28	-0.40
Cz19g02060	Hydroxymethylglutaryl-CoA synthase (HMG-CoA synthase)	*HCS*	1.12	-1.67	-0.54
Cz03g02150	Isopentenyl-diphosphate Delta-isomerase	*IPPI*	0.37	0.13	0.52
Cz02g31110	Geranyl diphosphate synthase	*GPPS*	0.32	-0.22	0.12
Cz01g03190	Farnesyl diphosphate synthase	*FPPS*	-0.82	0.63	-0.18
Cz02g19200	Geranylgeranyl diphosphate synthase	*GGPPS*	1.76	-1.81	-0.03
Cz05g32220	Phytoene synthase (chloroplastic)	*PSY*	5.62	-5.24	0.40

Cz02g32280	Phytoene desaturase	*PDS*	3.47	-2.64	0.86
Cz10g17010	Zeta-carotene desaturase (chloroplastic)	*ZDS*	2.22	-1.68	0.55
Cz10g17130	Zeta-carotene isomerase	*ZISO*	2.38	-1.74	0.65
Cz16g01210	Carotenoid isomerase	*CRTISO1*	3.33	-3.53	-0.17
Cz12g03260	Carotenoid isomerase	*CRTISO2*	-1.19	1.19	0.02
Cz14g22040	Carotenoid isomerase	*CRTISO3*	1.61	-1.33	0.29
Cz09g18310	Lycopene epsilon cyclase	*LCYe*	1.25	-1.86	-0.59
Cz12g10170	Lycopene beta cyclase	*LCYb*	0.70	-0.18	0.54
Cz13g16110	Cytochrome P450 beta hydroxylase,CYP97A5(Cr)	*CYP97A*	4.03	-3.57	0.48
Cz09g07100	Cytochrome P450 epsilon hydroxylase, CYP97C3	*CYP97C*	2.44	-2.76	-0.30
Cz09g14130	Cytochrome P450 CYP97B, beta or seta-rings (At)	*CYP97A*	0.74	-0.64	0.12
Cz12g16080	Beta-carotene hydroxylase	*CHYb*	0.45	-0.18	0.28
Cz13g13100	Beta-carotene ketolase/oxygenase	*BKT1*	1.23	-0.74	0.50
Cz04g11250	Beta-carotene ketolase/oxygenase	*BKT2*	0.95	-0.46	0.50
Cz07g30060	Zeaxanthin epoxidase	*ZEP*	2.18	-1.83	0.37
Cz06g02070	Violaxanthin de-epoxidase	*VDE*	-0.10	-0.28	-0.36
Cz02g29020	Long-chain-alcohol *O*-fatty-acyltransferase	*AAT?*	1.38	-1.04	0.36
**OPP Pathway**					
Cz06g12080	Glucose-6-phosphate 1-dehydrogenase	*G6PD1*	0.27	-0.45	-0.17
Cz03g12030	Glucose-6-phosphate 1-dehydrogenase	*G6PD2*	1.45	-1.46	-3.71
Cz04g18010	6-Phosphogluconolactonase	*PGLS1*	0.58	-0.05	0.55
Cz11g03250	6-Phosphogluconolactonase	*PGLS2*	-1.38	2.43	1.05
Cz05g06160	6-Phosphogluconate dehydrogenase	*6PGD*	-2.32	1.77	-0.53
Cz05g13260	Ribose 5-phosphate isomerase	*RPI1*	-5.36	5.45	0
Cz09g17220	Ribose 5-phosphate isomerase	*RPI2*	0.23	-0.57	-0.32
Cz05g11190	Ribulose-phosphate 3-epimerase	*RPE1*	2.33	-1.66	0.69
Cz04g31230	Ribulose-phosphate 3-epimerase	*RPE2*	1.15	-0.88	0.29
Cz14g07140	Ribulose-phosphate 3-epimerase	*RPE3*	2.33	-1.99	0.36
Cz03g04080	Transketolase	*TRK*	1.94	-2.01	-0.05
Cz06g25040	Transaldolase	*TAL1*	0.20	-0.82	-0.61
Cz04g12210	Transaldolase	*TAL2*	0.37	-0.15	0.24
Cz12g20290	Transaldolase	*TAL3*	-2.29	2.41	0.13
**Glycolysis and Glucogenesis**					
Cz13g07170	Hexokinase	*HK*	-0.73	1.32	0.60
Cz06g03010	Glucokinase	*GK*	-2.24	1.22	-1.00
Cz01g30020	Glucose-6-phosphate isomerase	*PGI*	-0.00	0.04	0.05
Cz09g25120	6-Phosphofructokinase	*PFK1*	-1.08	0.58	-0.48
Cz16g11260	6-Phosphofructokinase	*PFK2*	-1.36	2.49	1.14

Cz07g13120	6-Phosphofructokinase	*PFK3*	-1.54	0.93	-0.58
Cz04g03070	Fructose-1,6-bisphosphatase, class I	*FBP1*	2.52	-2.49	0.04
Cz05g01180	Fructose-1,6-bisphosphatase, class II	*FBP2*	0.71	0.91	1.63
Cz05g37140	Fructose-bisphosphate aldolase	*FBA1*	0.89	-1.05	-0.15
Cz06g07090	Fructose-bisphosphate aldolase	*FBA2*	0.11	-0.72	-0.59
Cz03g06050	Fructose-bisphosphate aldolase	*FBA3*	0.63	-0.46	0.19
Cz03g13070	Fructose-bisphosphate aldolase	*FBA4*	-2.42	1.66	-0.76
Cz06g17270	Triosephosphate isomerase	*TIM*	-0.28	0.18	-0.08
Cz05g34160	Glyceraldehyde 3-phosphate dehydrogenase (NAD)	*GAPDH*	-2.26	2.35	0.10
Cz16g19260	Phosphoglycerate kinase	*PGK*	0.38	-0.87	-0.47
Cz06g01110	Phosphoglycerate mutase, 2,3-bisphosphoglycerate-dependent	*PGAM1*	-2.81	2.28	-0.51
Cz09g03220	Phosphoglycerate mutase, 2,3-bisphosphoglycerate-independent	*PGAM2*	0.15	-0.29	-0.12
Cz04g11130	Phosphoglycerate mutase, 2,3-bisphosphoglycerate-independent	*PGAM3*	-3.66	3.38	-0.26
Cz08g21030	Phosphoglycerate mutase, 2,3-bisphosphoglycerate-independent	*PGAM4*	-0.86	-0.23	1.08
Cz14g01200	Phosphoglycerate mutase	*PGAM5*	0.66	-0.99	-0.32
Cz14g08090	Phosphoglycerate mutase	*PGAM6*	0.56	-0.50	0.07
Cz01g38050	Phosphoglycerate mutase	*PGAM7*	-0.95	1.35	0.41
Cz05g10010	Enolase	*ENO*	-1.64	1.20	-0.42
UNPLg00362	Pyruvate kinase	*PK1*	0.38	-0.68	-0.28
Cz08g12170	Pyruvate kinase	*PK2*	1.75	-1.90	-0.13
Cz16g00040	Pyruvate kinase	*PK3*	-0.79	0.68	-0.10
Cz14g14130	Pyruvate kinase	*PK4*	1.75	-1.26	0.50
Cz04g17050	Pyruvate kinase	*PK5*	-1.41	1.54	0.15
Cz01g21060	Pyruvate kinase	*PK6*	-1.42	2.24	0.82
Cz15g09100	Pyruvate kinase	*PK7*	-0.28	0.28	0.02
Cz10g06190	Pyruvate kinase	*PK8*	0.63	-1.10	-0.45
Cz01g05160	Phosphoenolpyruvate carboxykinase (ATP)	*PEPCK*	0.30	1.83	2.15
Cz04g02090	Pyruvate carboxylase	*PYC*	0.47	-0.21	0.28
**TCA**					
Cz02g27080	Citrate synthase	*CIS1*	0.08	0.94	1.03
Cz02g12210	Citrate synthase	*CIS2*	-3.90	2.57	-1.32
Cz13g00140	Aconitate hydratase	*ACH*	0.34	-0.31	0.05
Cz11g28180	Isocitrate dehydrogenase, NAD-dependent	*IDH1*	-2.66	1.96	-0.68
Cz11g08120	Isocitrate dehydrogenase, NAD-dependent	*IDH2*	-1.57	0.86	-0.70
Cz12g16160	Isocitrate dehydrogenase, NADP-dependent	*IDH3*	0.31	-0.70	-0.38
Cz05g03220	2-Oxoglutarate dehydrogenase, E1	*OGDH1*	-1.08	0.37	-0.69
Cz01g28100	2-Oxoglutarate dehydrogenase, E1	*OGDH2*	1.41	-0.95	0.48

Cz02g11010	2-Oxoglutarate dehydrogenase, E2	*OGDH3*	-1.84	0.75	-1.07
Cz01g33150	Succinyl-CoA synthetase, alpha subunit	*SCSa*	-1.20	0.22	-0.97
Cz03g31200	Succinyl-CoA synthetase, beta subunit	*SCSb*	-2.19	1.33	-0.84
Cz15g13230	Succinate dehydrogenase (ubiquinone) iron-sulfur subunit	*SDH1*	-0.83	1.14	0.32
Cz07g14020	Succinate dehydrogenase (ubiquinone) cytochrome b560 subunit	*SDH2*	-1.79	1.76	-0.01
Cz07g14015	Succinate dehydrogenase (ubiquinone) membrane anchor subunit	*SDH3*	-1.01	0.91	-0.08
Cz07g04030	Fumarate hydratase, class I	*FHD*	0.07	0.05	0.14
Cz02g21340	Malate dehydrogenase (NAD)	*MDH*	-2.45	1.25	-1.18
**Acetyl-CoA source**					
Cz18g13050	Pyruvate dehydrogenase, E1 alpha	*PDHC1*	-0.38	0.23	-0.13
Cz07g25130	Pyruvate dehydrogenase, E1 beta	*PDHC2*	-1.66	1.39	-0.25
Cz03g08090	Pyruvate dehydrogenase, E1 alpha	*PDHC3*	-0.72	0.72	0.02
Cz01g37230	Pyruvate dehydrogenase, E1 beta	*PDHC4*	-1.57	1.76	0.21
Cz05g28130	Pyruvate dehydrogenase, E2 (dihydrolipoamide acetyltransferase)	*PDHC5*	-3.26	2.97	-0.27
Cz10g25060	Pyruvate dehydrogenase, E3 (dihydrolipoamide dehydrogenase)	*PDHC6*	-0.48	0.25	-0.21
Cz07g16120	Pyruvate dehydrogenase, E3 (dihydrolipoamide dehydrogenase)	*PDHC7*	-0.35	0.45	0.11
Cz01g33100	Pyruvate decarboxylase	*PDC*	-0.25	-0.48	-0.72
Cz11g04290	Aldehyde dehydrogenase (NAD+)	*ALDH1*	-1.29	0.63	-0.64
Cz03g20090	Aldehyde dehydrogenase (NAD+)	*ALDH2*	-0.59	-0.09	-0.66
Cz05g18170	Aldehyde dehydrogenase	*ALDH3*	0.10	-0.61	-0.49
Cz09g15060	Acetyl-CoA synthetase	*ACS1*	-1.05	0.50	-0.53
Cz12g10100	Acetyl-CoA synthetase	*ACS2*	0.87	-0.12	0.77

Table S2 Amino acid composition of heterotrophic cultured *C. zofingiensis* after MP.

Amino acid	Composition (%)
Asp	9.97679
Ser	2.90932
Glu	14.18904
Gly	5.90862
His	2.96564
Arg	10.89968
Thr	2.01309
Ala	9.25401
Pro	6.02178
Cys	0
Tyr	0
Val	9.6075
Met	0
Lys	6.31571
Ile	5.60607
Leu	8.79234
Phe	5.54043
